# Acute glucoregulatory and vascular outcomes of three strategies for interrupting prolonged sitting time in postmenopausal women: A pilot, laboratory-based, randomized, controlled, 4-condition, 4-period crossover trial

**DOI:** 10.1371/journal.pone.0188544

**Published:** 2017-11-30

**Authors:** Jacqueline Kerr, Katie Crist, Daniela G. Vital, Lindsay Dillon, Sabrina A. Aden, Minaxi Trivedi, Luis R. Castellanos, Suneeta Godbole, Hongying Li, Matthew A. Allison, Galina L. Khemlina, Michelle L. Takemoto, Simon Schenk, James F. Sallis, Megan Grace, David W. Dunstan, Loki Natarajan, Andrea Z. LaCroix, Dorothy D. Sears

**Affiliations:** 1 Department of Family Medicine and Public Health, UC San Diego, La Jolla, California, United States of America; 2 San Diego State University, San Diego, California, United States of America; 3 Center for Clinical Research, Clinical and Translational Research Institute, UC San Diego, La Jolla, California, United States of America; 4 Department of Medicine, UC San Diego, La Jolla, California, United States of America; 5 Department of Orthopedic Surgery, UC San Diego, La Jolla, California, United States of America; 6 Baker IDI Heart and Diabetes Institute, Melbourne, Australia; 7 Mary MacKillop Institute for Health Research, Australian Catholic University, Melbourne, Australia; Garvan Institute of Medical Research, AUSTRALIA

## Abstract

**Background:**

Prolonged sitting is associated with cardiometabolic and vascular disease. Despite emerging evidence regarding the acute health benefits of interrupting prolonged sitting time, the effectiveness of different modalities in older adults (who sit the most) is unclear.

**Methods:**

In preparation for a future randomized controlled trial, we enrolled 10 sedentary, overweight or obese, postmenopausal women (mean age 66 years ±9; mean body mass index 30.6 kg/m^2^ ±4.2) in a 4-condition, 4-period crossover feasibility pilot study in San Diego to test 3 different sitting interruption modalities designed to improve glucoregulatory and vascular outcomes compared to a prolonged sitting control condition. The interruption modalities included: a) 2 minutes standing every 20 minutes; b) 2 minutes walking every hour; and c) 10 minutes standing every hour. During each 5-hr condition, participants consumed two identical, standardized meals. Blood samples, blood pressure, and heart rate were collected every 30 minutes. Endothelial function of the superficial femoral artery was measured at baseline and end of each 5-hr condition using flow-mediated dilation (FMD). Participants completed each condition on separate days, in randomized order. This feasibility pilot study was not powered to detect statistically significant differences in the various outcomes, however, analytic methods (mixed models) were used to test statistical significance within the small sample size.

**Results:**

Nine participants completed all 4 study visits, one participant completed 3 study visits and then was lost to follow up. Net incremental area under the curve (iAUC) values for postprandial plasma glucose and insulin during the 5-hr sitting interruption conditions were not significantly different compared to the control condition. Exploratory analyses revealed that the 2-minute standing every 20 minutes and the 2-minute walking every hour conditions were associated with a significantly lower glycemic response to the second meal compared to the first meal (i.e., condition-matched 2-hour post-lunch glucose iAUC was lower than 2-hour post-breakfast glucose iAUC) that withstood Bonferroni correction (*p* = 0.0024 and *p* = 0.0084, respectively). Using allometrically scaled data, the 10-minute standing every hour condition resulted in an improved FMD response, which was significantly greater than the control condition after Bonferroni correction (*p* = 0.0033).

**Conclusion:**

This study suggests that brief interruptions in prolonged sitting time have modality-specific glucoregulatory and vascular benefits and are feasible in an older adult population. Larger laboratory and real-world intervention studies of pragmatic and effective methods to change sitting habits are needed.

**Trial registration:**

ClinicalTrials.gov NCT02743286.

## Introduction

Population-based accelerometer studies show that older adults spend more time sitting during waking hours and are less likely to meet physical activity (PA) guidelines compared to other adult age groups [1]. Older adults also have the highest risk for type 2 diabetes, cardiovascular disease (including peripheral artery disease, hypertension, and coronary heart disease), cancer, and frailty, and these chronic conditions increase their mortality risk [2–4]. While it is well-established that PA is related to these conditions, a growing body of evidence suggests that sitting time also contributes to such outcomes, independent of time spent engaging in leisure-time PA [5–8]. In recognition of the emerging evidence, the American Heart Association’s has published a position statement on sedentary behavior, specifically calling for more specific quantitative evidence to inform public health recommendations and real-world interventions [9].

Recent laboratory studies manipulating specific behaviors have provided initial insights on the merits of sitting time interruption strategies that are acutely beneficial (i.e., within hours) and that can be tested in real world clinical trials. A 2015 review [10] found 14 acute laboratory experiments that compared prolonged sitting conditions with sitting interruption conditions. Sitting interruption modalities included standing, walking at different intensities, cycling, and resistance exercises. Frequency of interruptions ranged from every 20 minutes to once an hour. The review concluded that interruption of sitting time resulted in consistently favorable changes in postprandial metabolic parameters, particularly in those who had type 2 diabetes, were overweight/obese, and/or were physically inactive. Plausible biological mechanisms have been proposed for why frequently breaking up sitting may benefit health. Specifically, postural change that occur with standing up from a seated position immediately increases blood flow and hydrostatic pressure, particularly in the lower extremities [11, 12] as a result of gravitational force, and requires leg and lower trunk muscle contractions to raise the body and to sustain the standing position. The action of standing up also induces compensatory changes in blood pressure (BP), heart rate, and vascular tone within seconds. As such, it has been hypothesized that frequent transitions from the seated to standing position during waking hours could be favorable for glucose regulation, mitochondrial function, and endothelial function [13–20]. Specifically, increased blood flow increases shear stress and reduces oxidative stress in endothelial cells [15, 19, 20], enhances oxygen delivery to mitochondria in endothelial and muscle cells (myocytes), and enhances nutrient (e.g., glucose) and hormone (e.g., insulin) delivery to lower extremity myocytes. Increased muscle contraction stimulates intracellular signaling responses that should enhance mitochondrial activity in endothelial cells and myocytes and enhance insulin-dependent and–independent glucose uptake in myocytes. Furthermore, large muscle contractions, particularly those that occur when rising from a chair, are related to physical functioning, frailty and mortality [1, 21, 22]. Thus, postural changes, even with low energy expenditure, may be beneficial for health [1, 15, 17, 18, 22–26].

Most laboratory studies examining the impact of interrupting sitting time have included younger, active participants and employed interruptions involving PA or long bouts of standing (e.g. 45 minutes). Few studies have examined vascular outcomes that might be affected by brief postural changes, which are considered highly relevant in older adults who have increased cardiovascular risk. In preparation for a future randomized controlled trial, we designed this 4-condition, 4-period randomized, controlled, crossover laboratory trial to pilot test the feasibility of our protocol and to investigate the glucoregulatory and vascular outcomes of brief postural changes and brief bouts of PA in sedentary overweight/obese postmenopausal women. Sedentary overweight/obese postmenopausal women are the largest and fastest growing population of older adults and have significant cardiometabolic risk. Older women experience higher rates of mobility disability, consume more health care and require more long-term care services than older men [27–30]. However, no previous laboratory studies have focused specifically on this high-risk demographic group. In this study, we aimed to compare the acute metabolic and vascular endothelial function outcomes of a control prolonged sitting condition with three different conditions involving brief standing or walking interruptions of prolonged sitting. Our brief, low-intensity strategies for interrupting prolonged sitting were feasible and had modality-specific benefits on glucoregulatory and vascular outcomes in our study population of sedentary overweight/obese postmenopausal women.

## Materials and methods

### Study protocol

#### Overview

This randomized, 4-condition, 4-period crossover trial was approved by the University of California, San Diego (UCSD) Institutional Review Board. Participants provided written informed consent during the screening visit. The study was registered with Clinical Trials.gov (#NCT02743286). Participants attended 4 separate visits to the clinical laboratory to complete each trial condition in a randomized order. The conditions each entailed a 1-hr lead-in sitting phase followed by a 5-hr phase of prolonged sitting that included either a) a single midpoint bathroom break (control condition of prolonged sitting), b) 2 minutes of standing every 20 minutes, c) 2 minutes of walking every hour, or d) 10 minutes of standing every hour.

#### Recruitment and screening

Postmenopausal women aged 55 years and older who were overweight or obese (BMI 27–45 kg/m^2^) were recruited across a 6-week period during March and April of 2016. Recruitment occurred through multiple sources including UCSD research networks, clinics and listservs, and local community outreach. Participants were eligible if they self-reported to accumulate an average of at least 6 hours of sitting time per day, participated in less than 20 minutes of moderate-to-vigorous PA on less than 3 days per week, and had no menstruation for at least 1 year. Exclusion criteria were as follows: poor glycemic control [hemoglobin A1c (HbA1c) ≥ 53 mmol/mol (7%) in participants <65 years of age or HbA1c ≥ 58 mmol/mol (7.5%) in participants ≤65 years of age], anemia, type 1 diabetes, use of insulin medications, uncontrolled hypertension, personal or family history of venous thrombosis, use of tobacco or nicotine products, more than 5% weight change in the last 3 months, blood donation within 56 days of enrollment, chronic illness that could impact weight gain, regular use of immunosuppressant or corticosteroid medications, risk of thrombosis (defined as use of vasodilator medications along with a history of congestive heart failure, stroke, atrial fibrillation or more than 2 hospitalizations within 6 months of enrollment), current participation in a study related to PA or sedentary behavior, inability to walk for 5 minutes, inability to stand in place for 10 minutes at a time, or inability to stand from a seated position on their own.

**Fig 1:**
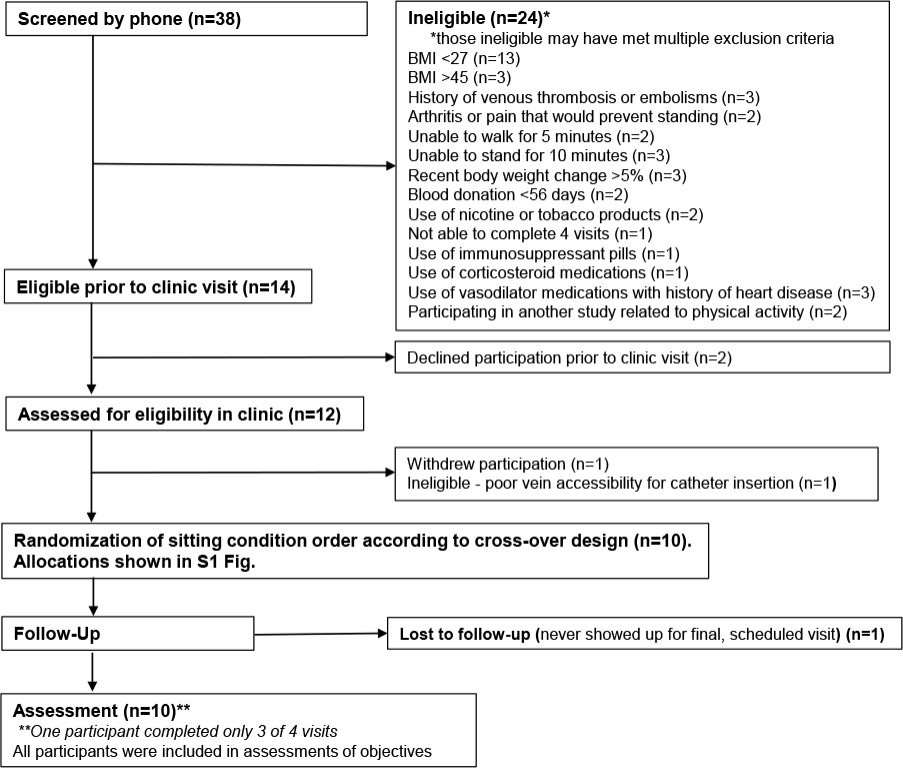
Consort diagram of enrollment and participation.

Potential participants were screened for eligibility by phone and, if eligible, sent a health history questionnaire to complete and scheduled for a medical screening at the UCSD Altman Clinical and Translational Research Institute Center for Clinical Research. At the screening visit, participants provided written informed consent. The Short Physical Performance Battery (SPPB) was performed to assess physical functioning and confirm participants’ ability to walk and stand without falling. Anthropometric measurements (height, weight, and waist and hip circumference), vital signs (BP and heart rate), and a 3-mL non-fasting blood sample were collected. Participants were screened for anemia using a complete blood count and for impaired glycemic control using HbA1c measurement, based on the criteria above. Fig 1 shows a CONSORT diagram of study recruitment, enrollment, and participation.

#### Study visits schedule and protocols

**Fig 2:**
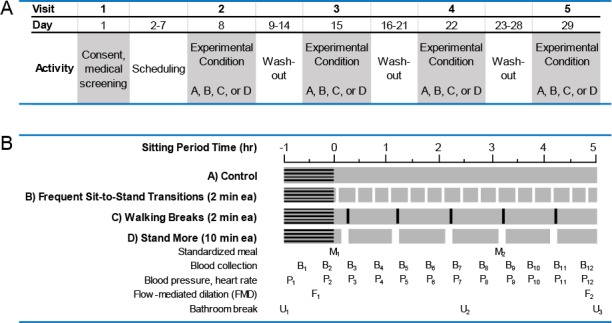
Sitting study design. Panel A. Schedule of participant study visits. Conditions A, B, C, or D were conducted in random order on Visits 2–5. Panel B. Schematic of sitting condition protocols’ activities. Horizontal black lines indicate the 1-hr lead-in sitting phase to achieve steady state. Vertical bars indicate sitting interruption events for respective conditions during the 5-hr sitting period. All conditions (A-D) included a single, per-protocol bathroom break at study period time 2.5hr. Sitting period time “0 hr” occurred immediately following P2 and B2, the moment when the breakfast standardized meal (M_1_) was initiated. M–standardized meal, B–blood collection, P–blood pressure and heart rate collection, F–FMD study, U–bathroom break.

Fig 2 shows the study visit schedule (Panel A) and experimental condition protocols (Panel B). Participants completed the 4 conditions in computer-generated random order. The randomization order of protocol conditions per participant is shown in S1 Fig. Participants were not informed of the condition they would follow until they arrived at each visit. Throughout the 4 conditions, participants watched DVDs, read books, magazines, or newspapers, performed light paperwork, or worked on a laptop computer. Study staff directly supervised participants throughout each study visit to ensure that full compliance with the sitting protocol conditions was achieved. A minimum wash-out of 7 days was required between each study visit to eliminate potential carryover effects and participants were asked to refrain from moderate-to-vigorous PA and consuming alcohol for 48 hours prior to study visits.

Participants were provided with a standardized dinner meal to consume the night before each study visit (450–475 kcal each, of which 23–25% were from fat, 52–56% were from carbohydrates, and 21–24% were from protein). They were asked to fast for at least 10 hours before arriving at the clinic and arrived between 0700 and 0830 hours by car or bus. Participants were permitted to take any required medications prior to the study visits if taken with water only or after the morning meal (M_1_ in Fig 2) if taken with food. Medication dosing was matched for all visits for each participant. Participants were asked to void their urine at the start of each visit and then were seated in a comfortably padded arm chair. A foot rest was provided if the participant’s feet did not comfortably rest flat on the floor. Initial BP and heart rate measurements were collected using an automated BP monitor (GE Carescape, Dinamap V 100; Alaris Critikon Dinamap PRO 300) at study period time -1.0hr. A catheter was inserted into an antecubital vein for blood sampling and an initial blood collection (B_1_) was collected 15 minutes after catheter placement (study period time −0.5 hr). Participants remained seated for the entire 1-hour lead-in time to achieve a steady state. Immediately following a second BP and heart rate measurement (P_2_) and blood draw (B_2_) at study period time 0 hr, participants consumed a standardized, liquid mixed meal (M_1_). Participants drank the liquid mixed meal (Ensure Plus®, 5 kcal/kg body weight) within 5 minutes at study period time 0 hr (“breakfast”, M_1_) and again at study period time 3 hr (“lunch”, M_2_). Calorie composition of the Ensure Plus® is 57% carbohydrate, 15% protein, and 28% fat. Water (8 oz) was provided after each meal. Mixed meals were used to simulate a typical postprandial glucose and insulin response to real-world meals across the day. Participants were guided through the study protocols for the remaining 5 hr.

Brachial arterial BP and heart rate measurements P_2_-P_12_ were collected every 30 minutes. Blood samples B_2_-B_12_ were collected every 30 minutes, immediately after BP and heart rate measurements. The timing of BP and heart rate measurement and blood sample collections was never less than 10 minutes after any sitting interruption. Endothelial function of the superficial femoral artery was directly assessed by flow-mediated dilation (FMD) at the start and end of each sitting protocol (details below).

#### Sitting condition protocols

As shown in Fig 2, the 4 conditions each included a 1-hr lead-in steady state period, a condition-variable 5-hr sitting period, and a per-protocol bathroom break at study period time 2.5-hr. The mid-protocol bathroom break occurred in a private restroom that was less than 20ft from the study room chair and was concurrent with a sitting interruption break during condition protocols B-D. The post-lead-in, 5-hr sitting condition protocols were as follows:

1. Control. Participants sat quietly for 5hr and were instructed to minimize excessive movement (i.e., fidgeting) while sitting. This condition was designed as the control condition for comparison in statistical analyses.
2. Frequent Sit-to-Stand Transitions. Participants completed 2 minutes of standing every 20 minutes throughout the 5-hr protocol period. In total, this condition amounted to 15 sit-to-stand transitions, 30 minutes of standing time, and a 10% reduction in sitting time compared to the 5-hr sitting control condition. This condition was designed to test the outcomes of frequent sitting interruptions consisting of short standing bouts.
3. Walking Breaks. Participants completed 2 minutes of light walking every hour throughout the 5-hr active protocol period. Participants were escorted during their walk in unobstructed clinic hallways and encouraged to walk at a comfortable, purposeful pace. In total, this condition amounted to 5 sit-to-stand transitions, 10 minutes of walking time, and a 3.3% reduction in sitting time compared to the 5-hr sitting control condition. This condition was designed to test the outcomes of hourly sitting interruptions consisting of short walking bouts that, if practiced across a whole day, would amount to meeting PA guidelines.
4. Stand More. Participants completed 10 minutes of standing every hour throughout the 5-hr active protocol period. Participants stood keeping their hips, legs and feet still on a modestly padded mat. They were permitted small, brief stretching movements of their upper body without lowering their head below waist level, and they had access to a standing desk to facilitate their continued work/reading/game activity while standing. In total, this condition amounted to 5 sit-to-stand transitions, 50 minutes of standing time, and a 17% reduction in sitting time compared to the 5-hr control condition. This condition was designed to test the outcomes of hourly sitting interruptions consisting of standing bout lengths that would be feasible in daily life for older adults and that, when practiced across the day, would amount to the greatest reduction in sitting time compared to the other interruption conditions. This condition was also designed to have the same number of sit-to-stand transitions as the Walking Breaks condition.

### Outcomes

#### Flow-mediated dilation

Endothelial function was assessed by femoral flow-mediated dilation (FMD) of the superficial femoral artery (SFA) at baseline and at the end of each study visit, per current guidelines [31] (Fig 2). FMD is a well-validated method used to assess endothelium-dependent vasodilation in response to an acute period of blood flow restriction and FMD is a sensitive predictor of atherosclerotic risk [32]. Arterial dilation is a healthy endothelial function response after release of acute blood flow restriction. The FMD procedure was conducted by an experienced vascular sonographer and analyzed by a board-certified physician with an expertise in ultrasound techniques. The FMD protocol was modeled after previously published studies [15, 19, 20]. Briefly, each FMD study was performed with the participant seated upright in the study chair, in a dark, quiet and climate controlled (22–25°C) condition. A 5 x 84 cm BP cuff was placed on the participant’s right thigh, 7–8 cm above the knee joint, distal to the site of ultrasound capture. Ultrasound images were obtained with a 2-D high-resolution ultrasound system (Phillips HD15 PureWave) using a 5–12 MHz multi-frequency linear-array transducer. The SFA was scanned with the ultrasound probe and a location with clear anterior and posterior intimal interfaces was selected. Landmarks were placed on the participant’s skin to ensure similar placement of the transducer for each of the 2 FMD studies conducted on the same day. In addition to imaging the arterial dimensions, Doppler ultrasound was used to measure SFA blood velocity as previously published [33].

SFA diameter images and Doppler measurements of blood velocity were continuously recorded for 5 seconds at baseline prior to cuff inflation. The BP cuff was then rapidly inflated 220–250 mmHg systolic BP and maintained for 5min until cuff deflation. SFA diameter and blood velocity recordings resumed at cuff deflation and continued for 5min. Ultrasound images were obtained throughout the 5 minutes of the FMD study and stored as “.avi” files. Off-line analysis of SFA diameters was performed using automated edge-detector software (Brachial Analyzer, Medical Imaging Applications, LLC, Coralville, IA, USA) as previously described [19]. This software allows the sonographer to determine the region of interest where the near and far vessel walls have the greatest clarity. The same vessel location on the participant was used for measurements throughout each study visit. Analyzed images were reviewed by the sonographer and edited as needed to ensure that diameter measurements were performed at an optimal location, intima-lumen interface at the near and far vessel wall. The peak SFA dilation after cuff deflation was presented as a percentage change from baseline diameter, % FMD: [(peak − baseline diameter)/baseline diameter×100] using raw data and after allometric scaling. Sitting protocol condition effects on FMD were assessed using a ratio of the end-of-study measurement FMD 2 (F_2_ in Fig 2B) divided by the baseline measurement FMD 1 (F_1_ in Fig 2B). FMD studies and outcomes assessments were conducted by investigators who were blinded to sitting condition protocol.

#### Biospecimen processing and plasma glucose and insulin measurement

Plasma was isolated from blood samples by centrifugation immediately after collection (1700xg for 10min at 4°C), aliquotted, and frozen at -80°C for assays on a later date. Plasma glucose concentration was determined using a YSI 2900D Biochemistry Analyzer® (Yellow Springs, OH) and plasma insulin concentration was determined using an ELISA assay per kit instructions (ALPCO catalog #80-INSHU; Salem, NH). All samples from a single participant were run on the same 96-well plate. Samples were distributed on 96-well plates in such a way as to minimize variance effects on data collection, e.g., loading order of protocol-specific sample sets was randomized. All assay plates included standard curve, normalization control, and quality control sample replicates (minimum of 2 per plate). The inter-assay coefficient of variance for the glucose assay was 6.2%; the inter-assay coefficient of variance for the insulin assay was 7.0%.

#### Blood pressure and heart rate

Systolic blood pressure (SBP), diastolic blood pressure (DBP), and heart rate measurements were collected using an automated BP monitor as described above in the *Study visits schedule and protocols* section. Readings were taken prior to blood sample collection at each collection time point.

#### Statistical analyses

This study was a pilot to test the feasibility of recruiting participants for the study, delivering the 4 sitting protocols and completing the intensive measurement regimen in postmenopausal women. Because it was a pilot study, no attempt was made to power the experiment to detect statistically significant differences in the various outcomes. However, our analytic methods test statistical significance within this small sample size. We used a mixed model to examine associations between condition and each of the outcomes, specifically, a random intercept model with a compound symmetry covariance structure. Eleven repeated measures of glucose, insulin, SBP, DBP, and heart rate collected during the 5-hr condition periods were used for analyses and graphed. For each participant and visit, the net incremental area under the curve (iAUC) of the 11 measures was calculated using a trapezoidal rule [34, 35], providing 4 iAUC estimates (one per condition) for each participant. Measurement #2 at time 0hr was used as baseline for iAUC calculations. These 4 iAUC values (one per condition) were the repeated measures outcome in the mixed models, with condition and baseline outcome level as fixed effects and subject-level random intercept. Statistical significance was assessed using Wald tests. In exploratory studies, separate iAUC values for the first 2 hours of each of the 2 post-meal periods (study period time intervals 0hr-2hr and 3hr-5hr) were also calculated and the above mixed model analyses was repeated for these period-specific iAUCs. The ratio of FMD 2 (afternoon measurement) to FMD 1 (baseline, morning measurement) was used to calculate condition-specific change in arterial dilation response to acute flow restriction. Mixed model analysis was conducted for FMD ratio (afternoon to morning). Statisticians were blinded to the interruption condition types during all analyses. Statistical software used was R. Bonferroni correction of *p*-values was applied to account for multiple testing in the 3- and 4-arm comparisons using a type 1 error cut-off of α < 0.05. Thus, as noted in respective figure legends, a statistically significant cut-off for 3-arm comparisons was p< 0.0167 (e.g., p<0.05/3) and a statistically significant cut-off for 4-arm comparisons was p< 0.0125 (e.g., p<0.05/4).

## Results

### Participant characteristics

Ten participants were enrolled in the study and nine participants completed all 4 sitting protocol conditions. One participant was lost to follow-up before her final study visit, thus, there are no data from the 2-minute standing every 20 minutes condition for this participant. On average, participants had abdominal obesity, as indicated by their BMI (mean >30kg/m^2^), waist circumference (mean >88cm), and waist-to-hip ratio (mean >0.85) (Table 1). On average, they also had impaired glucose regulation, as indicated by their elevated HbA1c levels (mean 5.7% or 39 mmol/mol, the lower limit of the “pre-diabetes” range), fasting plasma glucose concentration (mean >100mg/dL, the lower limit of impaired fasting glucose), fasting plasma insulin levels, and HOMA-IR values.

**Table 1:** Clinical characteristics (n = 10).

**Variable**		**Mean**	**Median**	**SD**
	Age (years)	66	65	9
	BMI (kg/m^2^)	30.6	29.3	4.2
	Body mass (kg)	79.4	76.8	12.3
	Height (cm)	161	160.8	5.9
	Waist circumference (cm)	95.9	94.5	11.8
	Hip circumference (cm)	104.5	105.7	15.2
	Waist/hip ratio	0.92	0.93	0.07
	HbA1c (%)	5.7	5.7	0.5
	HbA1c (mmol/mol)	39	39	6
	Fasting plasma glucose (mg/dL)*	107.2	102.2	17.4
	Fasting plasma insulin (uIU/mL)*	9.3	8.3	4.8
	HOMA-IR*	2.5	2.1	1.5
	Systolic pressure (mmHg)*	123	124	8
	Diastolic pressure (mmHg)*	66	65	7
	Heart rate (beats per minute)*	65	66	8
**Relevant Medications**		**Participant #**		
	β-blockers	1		
	β-3 adrenergic agonists	1		
	Calcium channel blockers	1		
	Loop diuretics	1		
	Metformin	2		
	Statins	2		
**Race**				
	White	9		
	Asian	1		
**Ethnicity**				
	Hispanic	2		
	Non-Hispanic	8		

* average at time "0" for all study visits, SD—standard deviation

### Sitting condition effects on postprandial plasma glucose and insulin concentrations

**Fig 3:**
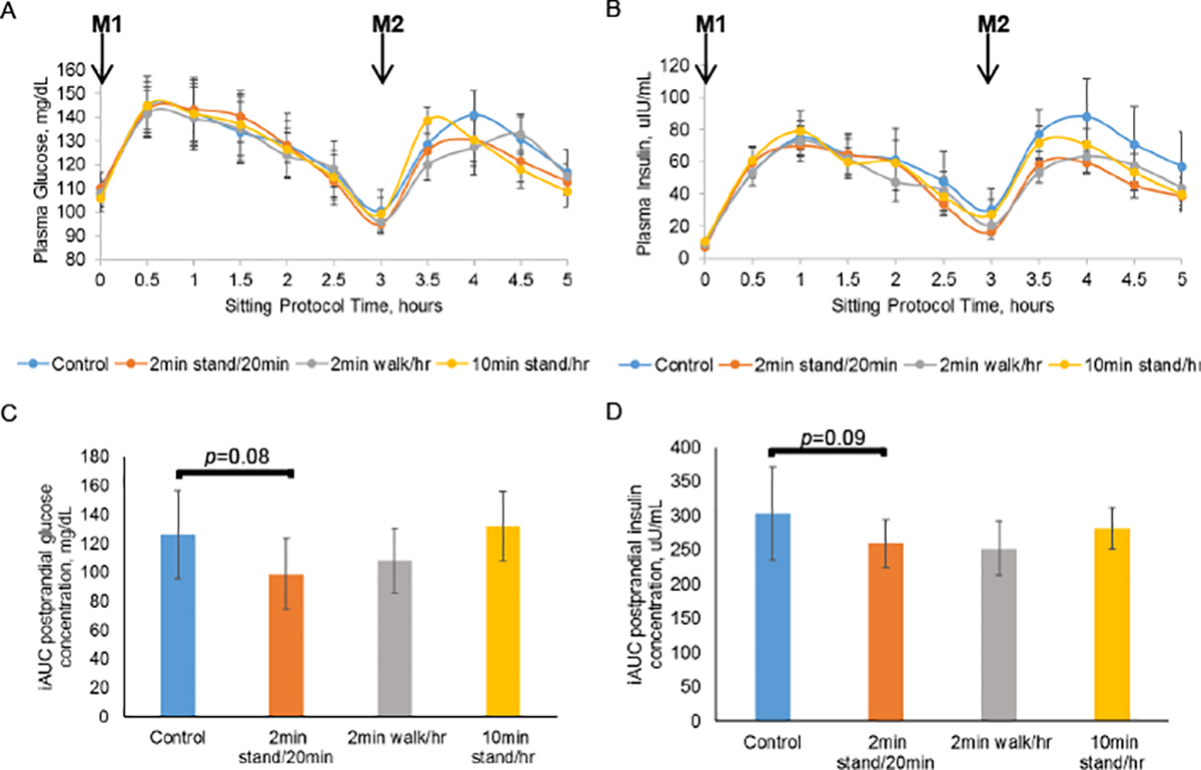
Glucose and insulin measurements during sitting conditions. Glucose (Panel A) and insulin (Panel B) concentrations at each time point. Average iAUC for postprandial glucose (Panel C) and insulin (Panel D) responses across the entire 5-hr sitting period for each interruption condition was compared to the control condition. Unadjusted p-values shown only for the comparison involving the 2-minute standing every 20 minutes condition. Bonferroni-corrected cut-off for significance in 3-arm comparison with control was *p*< 0.0167. All data shown are means +/- SEM; n = 10 for the control, 2-minute walking every hour, and 10-minute standing every hour conditions; n = 9 for the 2-minute standing every 20 minutes condition; M_1_ –breakfast meal, M_2_ –lunch meal.

This study was a pilot to test design feasibility and, although not powered to detect statistically significant differences in the various outcomes, our analytic methods test statistical significance within this small sample size. Generally, the glucose and insulin concentrations at each time point during the study period time interval of 2.5hr-5.0hr were lower in the 3 sitting interruption conditions compared to the control condition (Fig 3A and 3B). We did not observe any statistically significant differences in net incremental area under the curve (iAUC) for either glucose or insulin during the 0–5.0hr study period for any of the sitting interruption conditions compared to the control condition but there was a non-significant trend for reduced glucose and insulin iAUC (*p* = 0.08 and *p* = 0.09, respectively) for the 2-minute standing every 20 minutes condition (Fig 3C and 3D). Noting that postprandial glycemic responses to the second meal might be differentially affected by the sitting interruption conditions, we conducted exploratory analyses comparing the within-condition iAUC values during the first 2 hours of each of the 2 post-meal periods (study period time intervals 0hr-2hr and 3hr-5hr) for glucose and insulin. These exploratory analyses revealed that the 2-minute standing every 20 minutes and the 2-minute walking every hour conditions were each associated with a significant reduction in 2-hour glucose iAUC after the second meal (post-lunch glucose iAUC) compared to the condition-matched 2-hour glucose iAUC after the first meal, which withstood Bonferroni correction (*p* = 0.0024 and *p* = 0.0084, respectively) **S2 Fig**.

### Sitting condition effects on SFA FMD

**Fig 4:**
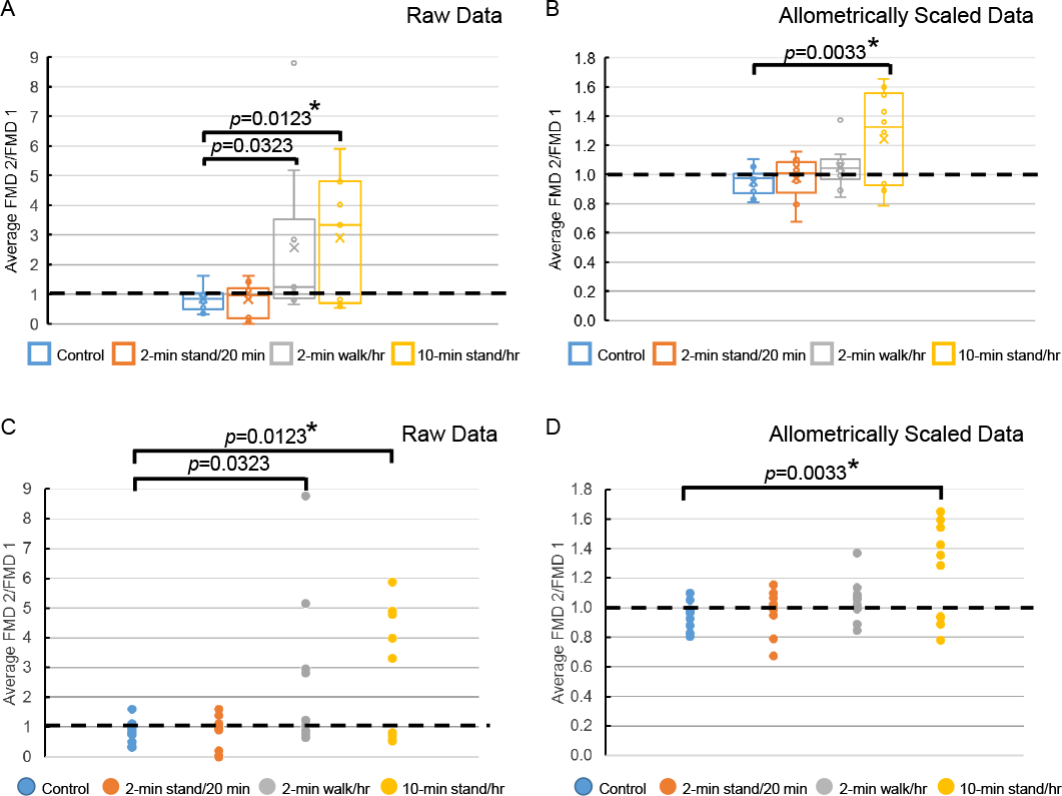
Change in FMD during sitting conditions. Condition-associated change in FMD is represented by the ratio of FMD 2 (end of sitting period) to FMD 1 (baseline) and are shown by group (Panels A and B) and by individual (Panels C and D) using raw data (Panels A and C) and allometrically scaled data (Panels B and D) (see Materials and Methods section). An FMD 2–to–FMD 1 ratio greater than 1 (dotted, horizontal line) indicates that the FMD response was greater at the completion of the sitting period relative to baseline. Box and whisker plots (Panels A and B): x = mean, line = median, dots above boxes are outliers. n = 10 for the control, 2-minute walking every hour, and 10-minute standing every hour conditions; n = 9 for the 2-minute standing every 20 minutes condition. *p*-value vs. the control condition. * Statistically significant after Bonferroni correction. Bonferroni-corrected cut-off for significance in 3-arm comparison with control was *p*< 0.0167.

Arterial dilation is a healthy endothelial function response after release of acute blood flow restriction. Change in dilation response reflects change in endothelial functioning. SFA FMD response was measured during each sitting condition protocol at baseline (FMD 1, morning measurement) and sitting period end (FMD 2, afternoon measurement) (see Fig 2B). The FMD 2/FMD 1 ratio was used to assess change in SFA dilation response between the two time points (Fig 4). An FMD 2/FMD 1 ratio greater than 1 (dotted, horizontal line) indicates that the SFA FMD response was improved at the completion of the sitting protocol relative to the beginning of the protocol. As there is some debate in the FMD literature about the importance of using allometric scaling, we report both raw data (Fig 4A and 4C) and allometrically scaled data (Fig 4B and 4D). Allometric scaling of FMD data controls for baseline diameter of the SFA which can be quite variable between individuals. On average, the 10-minute standing every hour condition resulted in an improved FMD response, which was significantly greater than the control condition using both raw data (p = 0.0123) and allometrically scaled data (p = 0.0033), even after Bonferroni correction. On average, the 2-minute walking every hour condition tended to improve the FMD response compared to the control condition when using the raw data, but this was not significant after Bonferroni correction and was eliminated after allometric scaling of the data.

#### Sitting condition effects on BP and heart rate

**Fig 5:**
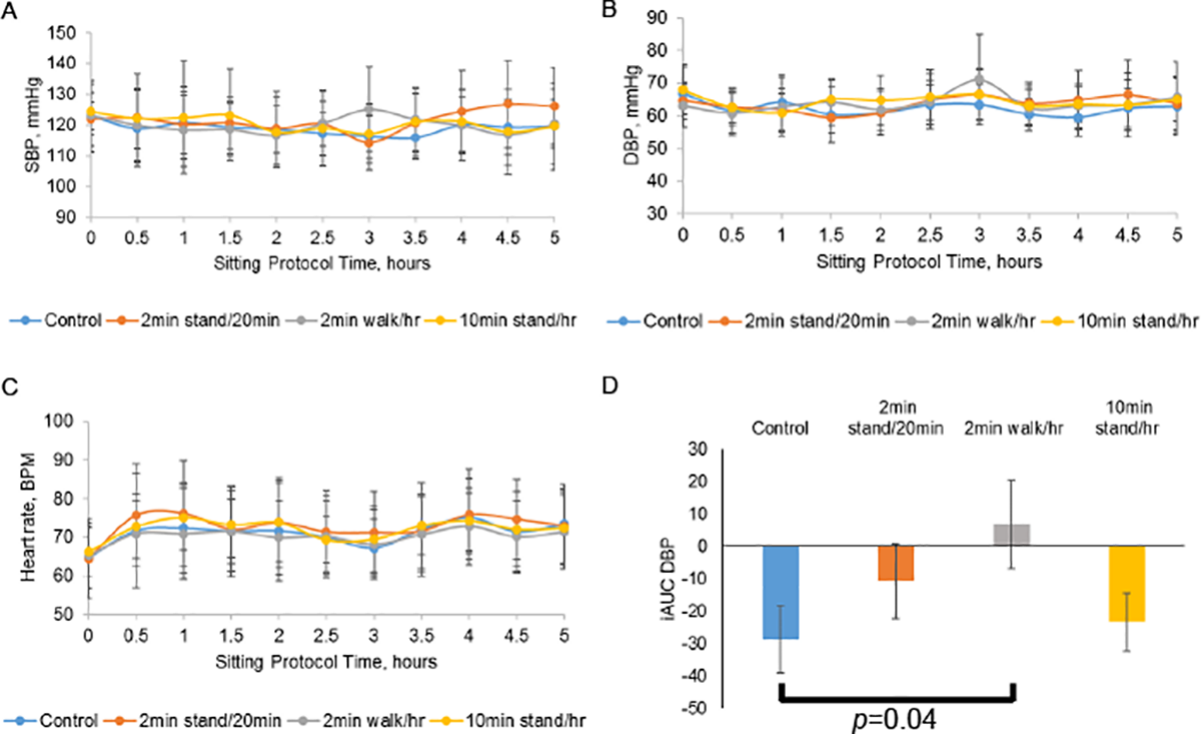
BP and heart rate during sitting conditions. SBP (Panel A), DBP (Panel B), and heart rate (Panel C) measurements at each time point. Panel D. Average iAUC for DBP values across the entire 5-hr sitting period. *p*-value vs. control condition. Bonferroni-corrected cut-off for significance in 3-arm comparison with control was p< 0.0167. All data are means +/- SEM; n = 10 for the control, 2-minute walking every hour, and 10-minute standing every hour conditions; n = 9 for the 2-minute standing every 20 minutes condition.

SBP, DBP, and heart rate measurements were collected every 30 minutes across the day (Fig 5). The average iAUC for DBP was nominally higher during the 2-minute walking every hour condition than during the control condition (Fig 5D, unadjusted *p* = 0.04) but this was not statistically significant after Bonferroni correction. DBP data from the two standing conditions were not different from control. None of the 3 sitting interruption conditions was different from control with respect to SBP or heart rate. There were no significant differences in outcomes related to the order in which the conditions were implemented at the clinic visits (see Fig 2A).

### Participant perceptions about sitting conditions and interruption modalities

Participants were surveyed about their perceived difficulty performing each sitting condition protocol and whether they thought that they could practice any of the 3 sitting interruption modalities in their daily lives. Using a scale of 0 to 4, (4 = easy, 3 = somewhat easy, 2 = neutral, 1 = slightly difficult, and 0 = very difficult), the average scores for the control, 2-minute standing every 20 minutes, 2-minute walking every hour, and 10-minute standing every hour conditions were 2.6, 3.4, 3.4, and 3.5, respectively. All the participants reported that they could or possibly could practice the 2-minute standing every 20 minutes and 2-minute walking every hour sitting interruption modalities on weekdays and weekend days in their daily lives. All but one participant reported that they could or maybe could practice the 10-minute standing every hour interruption modality on weekdays and weekend days in their daily lives.

## Discussion

We conducted a feasibility pilot study in preparation for a fully-powered randomized controlled trial in an at-risk population of aging, overweight/obese older women. This study was not powered to assess outcome measures but analytic methods were used to test statistical significance within this small sample size. Our pilot study design of 3 sitting interruption modalities incorporating brief standing and walking in sedentary, overweight postmenopausal women yielded results that were suggestive of modality-specific, positive impact on glucoregulatory outcomes and, with respect to the 10-minute standing every hour condition, significantly effective on vascular outcomes relative to prolonged sitting. Participants in our study had a mean age of 66 years and, on average, were obese with central adiposity and impaired glycemic control (Table 1). Thus, our study sample is reflective of a population that has significantly elevated risk for cardiovascular disease and for type 2 diabetes and its complications [2, 3]. Recruitment, protocol delivery, and measurement were all feasible and acceptable to study participants. This laboratory study, conducted in at risk, sedentary postmenopausal women, employed prolonged sitting interruption strategies that could be feasible in daily life for this age group.

Previous laboratory studies of glucoregulatory outcomes in participants who were generally younger and more active than participants involved in our study, showed that walking at various intensities were related to improved postprandial glucose and insulin responses compared to the control sitting condition [17, 18, 24, 25]. A recent study has shown postprandial glucoregulatory and lipid metabolism benefits in postmenopausal women practicing brief standing or walking breaks (5-minute breaks every 30 minutes’ of sitting)[36]. Other laboratory studies, conducted in participants younger and more active than those involved in our study as well as those who are overweight/obese and/or who have type 2 diabetes, have shown that low- to moderate-intensity walking breaks improve fatigue [37], endothelial dysfunction [15], and blood pressure [23, 26] outcomes associated with acute bouts of prolonged sitting. No previous study has tested interruptions as brief or as modest in intensity as those in our study design. Such brief, modest-intensity interruptions may be beneficial for older adults at high risk for CVD, but not other population groups. For example, in contrast to our study results, studies in young, non-obese participants showed that brief standing breaks in prolonged sitting did not lead to improved postprandial glycemic responses [25] and that brief walking breaks in prolonged sitting did not improve endothelial function but prevented sitting-induced impairment [15].

Although we did not observe any statistically significant, interruption condition-specific differences in postprandial glucoregulatory response for iAUC compared to the control condition in our pilot study, we did observe a trend for glucose and insulin iAUC improvement during the 2-minute standing every 20 minutes condition. In exploratory studies, both the 2-minute standing every 20 minutes and the 2-minute walking every hour conditions were associated with significant glucose-lowering during the second meal period (post-lunch glucose iAUC lower than post-breakfast iAUC). Our study design is a randomized controlled cross-over trial so, these meal period-specific impacts may be due to compounding benefits that the interruptions might have across the day, which manifest significantly after 3+ hours of practice. The start time of the post-lunch study period is associated with a 3-hour accumulation of 9 sit-to-stand transitions in the 2-minute standing every 20 minutes condition, 6 minutes of walking in the 2-minute walking every hour condition, and 30 minutes of standing time in the 10-minute standing every hour condition (Fig 2). Head down bed rest studies also suggest that duration, condition-type, and accumulated number of standing or walking bout interruptions are moderators of physiological benefit [38]. Additional studies in a larger sample size are needed to further explore this observation and possible mechanisms. Overall, our results suggest that regular interruptions of sitting throughout the day are needed to generate maximal glucoregulatory benefits. Our current study results are supported by a recent cross-sectional study of ours showing that great numbers of sit-to-stand transitions accumulated throughout the day are associated with lower fasting insulin and lower insulin resistance a similar demographic population [39]. Our current study sample (sedentary, older, overweight or obese women) are at high risk for cardiovascular disease and for type 2 diabetes and its complications; thus, any method that improves postprandial glucose and insulin responses in this population could have clinically meaningful results if practiced regularly.

We also observed that the 10-minute standing every hour condition resulted in acutely improved endothelial functioning compared to the control condition, using both raw and allometrically scaled data. This suggests that reducing sitting time by only 17% can acutely improve vascular function in a sedentary population with high cardiovascular risk. Further, it indicates that extended postural change, e.g., standing for 10 minutes, but not shorter periods of postural change, e.g., walking or standing for only 2 minutes per bout, can improve SFA FMD. One possible mechanism by which FMD is improved with 10-minute standing every hour is through extended compensatory changes in blood pressure (BP) and vascular tone that occur with standing that would persist during each standing bout. Additionally, standing bout-induced gravitational blood flow would increase shear stress which can reduce oxidative stress in endothelial cells [15, 19, 20] and result in improved FMD. Additional studies are needed to determine the cellular and physiological mechanisms by which acute interruptions of prolonged sitting benefit this study population. Thosar, et al. and others [15, 19, 20] have shown that endothelial functioning, also assessed by SFA FMD, is acutely damped by a 3-hour bout of prolonged sitting and that light walking interruptions (5 minutes at 2 miles per hour each hour) during this sitting time prevent that endothelial dysfunction. We found no evidence that a 5-hour bout of prolonged sitting leads to impaired endothelial dysfunction in our pilot study population. Previous studies, however, enrolled a sample of healthy, active young men, different from our sample of overweight or obese, older-age women. Extended sitting might be less routine in younger, active populations and, therefore, more detrimental to them. In our population of women who each regularly sit for 6 or more hours, no detrimental impact was observed. We specifically selected our population sample because it represents those in greatest need of feasible and effective sitting interruption modalities to improve cardiometabolic aspects of healthy aging [27–30].

Significantly elevated DBP observed in the 2-minute walking every hour condition compared to the control condition may be the residual effect of a normal, PA-induced increase in BP that does not rebound quickly in our sample of sedentary older women. One study of overweight/obese adults (average age 53.8 years) observed that a sitting interruption of 2 minutes light- or moderate-intensity walking every 20 minutes resulted in decreased SBP and DBP compared to a control sitting condition [26]. The women in our study, who normally sit for many hours each day, did not exhibit adverse BP effects during the control prolonged sitting condition, whereas young active group in the aforementioned study was adversely affected. Again, this difference may be due to differences in blood vessel functioning between the two study populations, sample size, or to protocol design.

Overall, our results suggest that healthy aging-related benefits associated with improved vascular function and glucose regulation can be realized acutely by breaking up prolonged sitting time with simple, practical interruptions including frequent, brief sit-to-stand transitions, hourly brief walking breaks, and hourly replacement of sitting time with short bouts of standing. Nearly all participants in our study thought they could practice each of these sitting interruption modalities in their daily lives. A key finding from our study is that the 10-minute standing every hour condition led to improved endothelial functioning compared to the control condition. In addition, exploratory studies showed that the 2-minute standing every 20 minutes condition and the 2-minute walking every hour condition significantly reduced the glycemic response to the second meal. This pilot study suggests that different sitting interruption modalities will have different vascular and glucoregulatory outcomes. Similarly, head down bed rest studies demonstrate that standing and walking breaks can have differential benefits that vary by physiological outcome measure and that are more pronounced with accumulated exposures [38]. Feasibility, vascular, and suggestive glucoregulatory outcomes of this pilot study support the need for fully-powered, randomized controlled trials in this population. In general, larger laboratory and real-world intervention studies of pragmatic and effective methods to improve health outcomes through sitting habits change are needed in older adults, men and women, and in age groups across the life-span and activity levels. Furthermore, given that each condition in our study affected different outcomes to different degrees, multiple mechanisms, i.e., both glucoregulatory and vascular, should be explored concurrently in future studies.

## Supporting information

S1 Fig Randomization order of protocol conditions per participant. (PDF)

S2 Fig Exploratory analyses of first and second meal postprandial glucose and insulin. Postprandial glucose (Panel A) and insulin (Panel B) iAUC averaged across the initial 2-hr post-breakfast period (0hr-2hr, diagonal stripe bars) and the 2-hr post-lunch period (3hr-5hr, horizontal stripe bars). n = 10 for the control, 2-minute walking every hour, and 10-minute standing every hour conditions; n = 9 for the 2-minute standing every 20 minutes condition. p-value for within-condition comparison of 2-hr post-lunch period vs. the 2-hr post-breakfast period. * Statistically significant after Bonferroni correction. Bonferroni-corrected cut-off for significance in 4-arm comparisons was p< 0.0125. ns–not significant.
(PDF)

S1 File CONSORT checklist pilot. (PDF)

S2 File Approved research plan UCSD IRB #150509. (PDF)
