# Supplementary material for: Acute glucoregulatory and vascular outcomes of three strategies for interrupting prolonged sitting time in postmenopausal women: A pilot, laboratory-based, randomized, controlled, 4-condition, 4-period crossover trial
Source: PLoS One. 2017 Nov 30;12(11):e0188544. doi: 10.1371/journal.pone.0188544 (PMC5708739; doi:10.1371/journal.pone.0188544)
Supplement: S1 Fig — (PDF) [file pone.0188544.s001.pdf]

S1 Fig. Randomization Order of Protocol Conditions Per Participant

	SB001	SB002	SB004	SB005	SB007	SB009	SB010	SB011	SB012	SB013
Visit 2	D	D	A	D	B	B	B	A	C	D
Visit 3	A	B	D	B	D	A	C	B	A	A
Visit 4	B	C	C	C	A	D	A	D	B	B
Visit 5	C	A	B *	A	C	C	D	C	D	C

Conditions: A – Control, B – Frequent Sit-to-Stand Transitions (2 min ea), C – Walking Breaks (2 min ea), D – Stand More (10 min ea), * participant lost to follow-up, never showed up for scheduled visit.
