# Supplementary material for: Acute glucoregulatory and vascular outcomes of three strategies for interrupting prolonged sitting time in postmenopausal women: A pilot, laboratory-based, randomized, controlled, 4-condition, 4-period crossover trial
Source: PLoS One. 2017 Nov 30;12(11):e0188544. doi: 10.1371/journal.pone.0188544 (PMC5708739; doi:10.1371/journal.pone.0188544)
Supplement: S2 Fig — Postprandial glucose (Panel A) and insulin (Panel B) iAUC averaged across the initial 2-hr post-breakfast period (0hr-2hr, diagonal stripe bars) and the 2-hr post-lunch period (3hr-5hr, horizontal stripe bars). n = 10 for the control, 2-minute walking every hour, and 10-minute standing every hour conditions; n = 9 for the 2-minute standing every 20 minutes condition. p-value for within-condition comparison of 2-hr post-lunch period vs. the 2-hr post-breakfast period. * Statistically significant after Bonferroni correction. Bonferroni-corrected cut-off for significance in 4-arm comparisons was p< 0.0125. ns–not significant. (PDF) [file pone.0188544.s002.pdf]

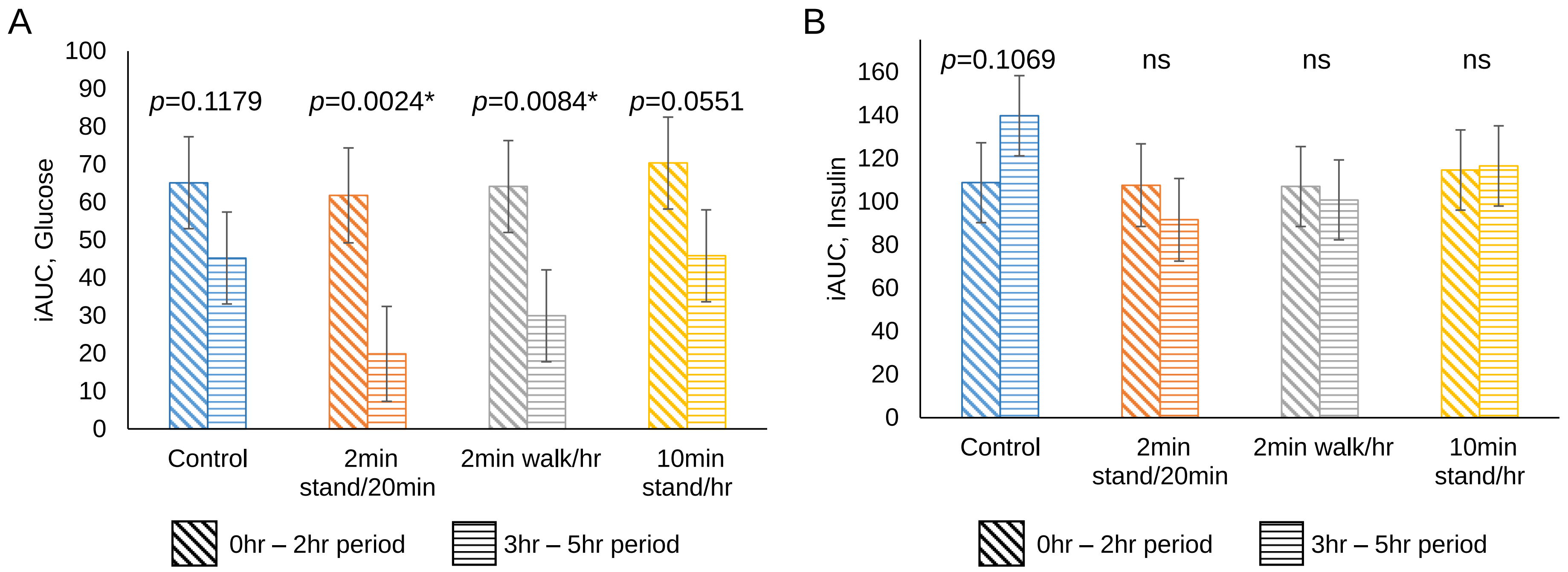

S2 Fig. Exploratory Analyses of First and Second Meal Postprandial Glucose and Insulin. Postprandial glucose (Panel A) and insulin (Panel B) iAUC averaged across the initial 2-hr post-breakfast period (0hr-2hr, diagonal stripe bars) and the 2-hr post-lunch period (3hr-5hr, horizontal stripe bars). n=10 for the control, 2-minute walking every hour, and 10-minute standing every hour conditions; n=9 for the 2-minute standing every 20 minutes condition. *p*-value for within-condition comparison of 2-hr post-lunch period vs. the 2-hr post-breakfast period. * Statistically significant after Bonferroni correction. Bonferroni-corrected cut-off for significance in 4-arm comparisons was $p < 0.0125$. ns – not significant.
