# Supplementary material for: Acute glucoregulatory and vascular outcomes of three strategies for interrupting prolonged sitting time in postmenopausal women: A pilot, laboratory-based, randomized, controlled, 4-condition, 4-period crossover trial
Source: PLoS One. 2017 Nov 30;12(11):e0188544. doi: 10.1371/journal.pone.0188544 (PMC5708739; doi:10.1371/journal.pone.0188544)
Supplement: S2 File — (PDF) [file pone.0188544.s004.pdf]

UCSD Human Research Protections Program
New Biomedical Application
RESEARCH PLAN

Instructions for completing the Research Plan are available on the HRPP website.
The headings on this set of instructions correspond to the headings of the Research Plan.
General Instructions: Enter a response for all topic headings.
Enter "Not Applicable" rather than leaving an item blank if the item does not apply to this project.

Version date: 9/30/2013

1. PROJECT TITLE

Sedentary Behavior Interrupted - A Pilot Study of Acute Interventions on Prolonged Sitting – IRB#150509

2. PRINCIPAL INVESTIGATOR

Dorothy D. Sears, Ph.D.
Associate Adjunct Professor of Medicine
Division of Endocrinology & Metabolism
9500 Gilman Drive, MC0673
University of California, San Diego
Phone: (858) 534-8898
Fax**:** (858) 534-6653

3. FACILITIES

UC San Diego, CTRI Center for Clinical Research (CCR)
9444 Medical Center Drive
La Jolla, CA 92093
Phone: (858) 657-5165
Fax: (858) 657-5059

4. ESTIMATED DURATION OF THE STUDY

Approximately 3 months

5. LAY LANGUAGE SUMMARY OR SYNOPSIS (no more than one paragraph)

Sedentary behavior, characterized by excess sitting time during waking hours, is detrimental to health and increases cardiometabolic disease (e.g., type 2 diabetes) risk, independent of moderate-to-vigorous physical activity. The mechanisms that mediate this are unknown and there are no evidence-based methods known for effectively intervening on sedentary behavior. The consequences of prolonged sitting time are of particular interest in older adults as sedentary behavior and cardiometabolic disease risk both increase with aging and moderate-to-vigorous physical activity may not be feasible. We propose pilot and feasibility testing of interventions for sedentary older adults that are designed to increase sit-to-stand transitions during prolonged sitting time. Interrupting sitting time through sit-to-stand transitions, and standing and walking breaks increase muscle use and blood flow in the lower parts of the body. Thus, we believe that frequent sit-to-stand interruptions of sitting time are the most efficacious sedentary behavior interventions, compared to simply reducing sitting time or less frequent walking breaks, for improving health outcomes and healthy aging. **We hypothesize that frequency of sit-to-stands during a 5-hour sitting period will result in health benefits that can be observed with a simple 2-minute standing interruption, and that this will be associated with improvements in metabolism and endothelial function**. This **pilot**, 10-participant study will 1) generate preliminary data for a Program Project Grant application that we are re-submitting to the National Institute of Aging that focuses on postmenopausal women, the fastest growing aged population with high life-time risk of cardiometabolic dysfunction and risk. This pilot study will inform our selection of sitting interruption modalities for two projects in our Program Project Grant application: "Project 1: Sedentary Behavior Interrupted: A randomized crossover treatment trial of acute effects on biomarkers of healthy aging in the laboratory (86 participants)" and "Project 2: Sedentary Behavior Interrupted: A randomized trial of 6 month effects on biomarkers of healthy aging and physical functioning in the real world (660 participants)." The current design of this pilot study is enhanced by and responsive to feedback from reviewers in response to our initial NIH/NIA submission. Importantly, this pilot study will increase our knowledge about how sedentary behavior and sitting interruption interventions influence healthy aging in postmenopausal women.

6. SPECIFIC AIMS

The deleterious health consequences of too much sitting are independent of moderate-to-vigorous physical activity. Understanding the health consequences of sitting time is particularly relevant for older adults who are extremely sedentary and at high risk of several chronic diseases that have been linked with sitting time, including type 2 diabetes. Interestingly, sedentary behavior is least studied among older adults who may benefit the most from reducing sitting time. Reducing uninterrupted sitting time in older adults is a research priority because the pervasiveness of sitting time, typically >8 hours per day, provides many opportunities for intervention. Specific interventions are required to reduce sitting because it is such a habitual behavior and is reinforced by social norms and chair-dominant contexts.

In this pilot study, we focus three different types of prolonged sitting interruptions as variables. Studies have shown that frequent breaks in sitting may reduce risk for cardiometabolic disease. In older adults, we anticipate that frequent sit-to-stand transitions will impact metabolic, inflammatory and other biomarkers, in particular those related to mitochondrial and endothelial function. A key limitation of previous sedentary intervention studies is that they have not impacted frequency of sit-to-stand transitions; rather, they decreased total sitting and increased standing time. Further, previous sedentary intervention studies have not directly compared frequent sit-to-stand transition interruptions with other interruption modalities associated with less frequent sit-to-stand transitions, for example, reduced overall sitting time (standing more) or with occasional walking breaks. We propose to test these sedentary behavior interruptions modalities to disrupt prolonged sitting and explore mitochondrial- and endothelial function-based mechanisms of action. Biologic theories suggest that interrupting sitting via sit-to-stand muscle contraction and increased blood flow to extremities is key to improving in healthy aging outcomes.

In a controlled laboratory study of prolonged sitting, we will conduct 3 different interruption break types. We will enroll 10 sedentary, postmenopausal participants 55+ years of age and body mass index (BMI) of 27-45. After screening and enrollment, participants will visit the clinic on 4 occasions for a 6-hour monitoring period and complete each of 4 5-hour sitting protocols following a 1-hour sitting run-in period. See **Figure 2** in the Research Design section for schematic of protocols. The 3 sitting interruption protocols will each include sitting interruptions of different break frequency and type, allowing us to study the role of sit-to-stand transitions and interruption type on acute metabolism and health outcomes and evaluate sitting interruption intervention efficacy. All study protocols include baseline, mid-point, and end-of-study bathroom breaks for urine sample collection with additional bathroom breaks allowed as needed. During each visit at multiple time points, we will collect blood to assess changes in biomarkers of mitochondrial function and meal tolerance (glucose and insulin areas under the curve) over the course of the sitting protocols. We will also collect measures of endothelial function, specifically, blood pressure (indirect measure) and flow-mediated dilation (direct measure). We will bank a small aliquot (0.5mL) of plasma samples and also collect and bank urine samples during the protocol for possible ancillary, exploratory studies. Participants may opt out of the sample banking. We will store de-identified urine and plasma samples in the Sears laboratory freezer for up to 2 years for possible ancillary studies. Our study is a crossover design and each participant will be her own control. **We hypothesize that frequency of sit-to-stands during a 5-hour sitting period will result in health benefits that can be observed with a simple 2-minute standing interruption, and that this will be associated with improvements in metabolism and endothelial function**. We will test this hypothesis in the following aim:

Study Aim: Using a randomized, controlled crossover study design, assess the feasibility and metabolic and endothelial function outcomes of an acute bout of prolonged sitting ± standing or walking interruptions using targeted metabolic and endothelial function biomarker analyses. We will enroll 10 postmenopausal women for this pilot study. We will measure plasma levels of insulin, glucose, and targeted mitochondrial metabolites including amino acid and lipid metabolites, especially branched chain amino acids and acyl-carnitines, and blood pressure at specific time points throughout each participant's 4 study clinic visits including fasting baseline and meal tolerance test (MTT) postprandial states. We will also collect measures of endothelial function, specifically,

blood pressure (indirect measure) and flow-mediated dilation (direct measure).

7. BACKGROUND AND SIGNIFICANCE

BACKGROUND

Association between sitting and chronic disease risk. Sitting time is dangerous for one's health and especially with respect to chronic diseases associated with aging. Sitting time is directly linked to obesity, cardiovascular disease, type 2 diabetes, and all cause mortality. Although the detrimental effects of sedentary behavior affect people of all ages, even pre-teen children,[1] middle-aged and older adults have increased degrees of sedentary behavior and its impact is more significant because of the independent influence of older age on these detrimental health effects. A recent study in men and women with type 2 diabetes or pre-diabetes, average age of 65 and average BMI of 31, showed that total sitting time was more closely related to BMI than total physical activity time.[2] These investigators and others in the fields of public health and physical activity research suggest that lifestyle interventions to decrease the risk of obesity and type 2 diabetes should aim not only at increasing total physical activity time, but also at reducing the total sitting time. Several studies show that total sitting time predicts risk for obesity independent of physical activity time.[3 4, 5] A large study of >63,000 Australian men age 45-64 showed that sitting time was significantly associated with type 2 diabetes and overall chronic disease, independent of physical activity and BMI.[6] Interestingly, increased physical activity in the absence of reduced sitting time does not negate the negative health effects of sedentary behavior[7] and women who participate in recommended physical activity time do not sit less than those not participating in physical activity.[8] Physical activity increases energy expenditure, blood flow and muscle contractions and these effects partly explain the positive relationship between physical activity and health. Single bouts of physical activity may not mitigate the effects of extended sitting throughout the day but short bouts of physical activity (e.g., 3 walking breaks per hour of 2 minutes each, **Figure 2**) might be effective.

Reducing sitting time. Reduction of sitting time is now clearly a public health focus.[9] However, solely reducing total sitting time is not the complete solution. Increasing evidence shows that reducing sitting time with more frequent interruptions is better for reducing metabolic and cardiovascular risk factors than the same reduction of sitting time with longer, less frequent interruptions.[10, 11] Several laboratory-based studies have shown that frequent, low-intensity activity breaks improves both postprandial glucose and insulin excursion during the course of a day of extended total sitting time.[12, 13] They demonstrate that frequent, low-intensity activity breaks of sitting time were as effective as frequent, moderate-intensity activity breaks[12] and metabolically more beneficial than a single 30min bout of exercise prior to the extended sitting period.[13] These and other acute extended sitting studies have enrolled only non-obese, healthy, young-to-middle aged adult participants. Laboratory studies of obese, older adult participants are needed. In addition, biomarkers that will better elucidate mechanistic pathways should be included in laboratory studies. We plan to study older postmenopausal women participants and a broader scope of biomarkers than has been published previously. Not only will our studies provide new mechanistic insights regarding this particular sector of the population with respect to their increased risk for detrimental effects of sitting but, in addition, older adults are likely to experience greater incremental benefit of sedentary time intervention compared to that observed in young-to-middle aged adults.[12, 13] The Diabetes Prevention Program is a precedent for such an effect as moderate lifestyle intervention was significantly more effective at reducing incident type 2 diabetes risk in older adults (71% reduction) than in the overall population (58% reduction).

Unanswered questions regarding extended sitting time – intervention guidelines. Health professionals and public health advocates need more evidence-based information regarding what sitting intervention guidelines to recommend to the public. How much sitting time is too much? How often and in what manner should one interrupt their sitting time? Does simply standing up suffice or should one walk about and stretch during sitting interruption? Are sitting interruption interventions feasible? These questions need to be answered by future research in real-world randomized controlled intervention studies and laboratory-based studies, such as we propose in this current research plan (pilot, laboratory-based study) and which we are proposing in a 2016

revised Program Project Grant application to the National Institute of Aging (proposal includes a laboratory-based project, a randomized controlled trial, and a large longitudinal cohort study).

Unanswered questions regarding extended sitting time – mechanisms/metabolism. Published laboratory studies described above[12, 13] have shown that regular interruption of extended sitting time improves postprandial glucose and insulin responses in young-to-middle aged, non-obese, healthy adults. The mechanisms by which regular sitting time interruptions confer this metabolic benefit are not known. Hamilton and colleagues have found in a rat model that physical inactivity is associated with suppression of skeletal muscle lipoprotein lipase activity.[14] They have also shown in rats and healthy, fit humans that expression of phosphatidic acid phosphatase type 2A (aka, LPP1/PAP2A) is decreased during physical inactivity.[15] Changes in activity/expression of these two enzymes occur within hours of initiating physical inactivity. As they are both involved in skeletal muscle lipid uptake and metabolism, we expect to observe that plasma lipids and their metabolites will be affected by our sitting protocols. Sit-to-stand transitions engage large muscle groups in the legs and trunk and activate mitochondrial metabolic pathways for ATP generation including oxidative phosphorylation, fatty acid oxidation, and Krebs cycle. Newgard, Gerszten, and others have elegantly demonstrated that specific mitochondrial pathways are dysfunctional in obesity (e.g., lipid oxidation and branched chain amino acid catabolism),[16] that plasma concentration of intermediary metabolites of these pathways is significantly altered as a result, and further, that these metabolites are biomarkers of insulin resistance[17] and predict type 2 diabetes years before onset.[18, 19] Dr. Sears (the PI) and others have demonstrated the utility of using "omics" data and systems biology approaches to characterize complex metabolic states and flux associate with insulin resistance and therapeutic intervention.[20-22] We expect that the detrimental effects of extended sitting are associated with mitochondrial dysfunction and that the mitochondrial metabolite biomarkers characterized by Newgard and Gerszten will be affected by our sitting protocols. We will use a targeted metabolomic profiling, mass spectrometry approach similar to that of Newgard[23] for this sitting study which includes eight free fatty acids, 15 amino acids, and 45 acyl-carnitines. Overall, the mechanisms that we will focus on in this proposed study are based on the evidence above and involve the functionally interwoven outcomes of defective insulin action, mitochondrial nutrient metabolism (amino acids, lipids), and lipid uptake.

Unanswered questions regarding extended sitting time – mechanisms/endothelial dysfunction. Extended sitting influences several biological mechanisms, including postural blood flow, energy expenditure, and muscle contraction. Throughout many hours across the day, extended sitting reduces postural blood flow to the lower extremities, energy expenditure, and skeletal muscle contractile activity. These physiological perturbations are the mechanisms that lead to changes not only in biomarkers of glucose regulation and mitochondrial function but also endothelial function.[24-27] Controlled studies have established that blood pressure, flow-mediated dilation (FMD), and other aspects of endothelial function are influenced by prolonged sitting.[24,25,28,29] Bed rest and space flight studies focus on glucose regulation, mitochondrial function, and endothelial function.[30,31] These types of studies are relevant to our project as they physiologically mimic the aging process.[31] A few studies of uninterrupted versus interrupted sitting have included biomarkers of endothelial functioning and actual endothelial function including blood pressure, hemostatic parameters, femoral FMD, and blood flow and shear rates. [24,25] Interestingly, antioxidant Vitamin C acutely prevents lower extremity decline in endothelial function during 3 hr of sitting, suggesting that oxidative stress may contribute to endothelial dysfunction during sitting.[24]

There is a need to study biomarker outcomes in crossover studies of sitting interruptions that are relevant to postural changes. We have designed a 4-period 4-condition randomized crossover clinical laboratory study during which we will assess the ability of standing and walking types of interruptions to impact the negative effects of prolonged sitting. The physiological mechanisms activated by standing (postural blood flow, energy expenditure, and muscle contraction) will improve biomarkers of glucose regulation, mitochondrial function, and endothelial function. No laboratory studies of acute changes associated with prolonged sitting interrupted

by standing or walking breaks in a crossover trial have included concurrent measurements of circulating biomarkers of metabolism or mitochondrial functioning nor clinical measures of endothelial functioning. As such, our study design is unique in concurrently assessing outcomes of acute glucose regulation, mitochondrial function, and endothelial function.

Study population – postmenopausal women. We will study postmenopausal women in this pilot study and in our proposed program project. Postmenopausal women are the fastest growing age/sex population subset in the United States. Postmenopausal women experience a high prevalence of metabolic disturbances; prior to, during, and post-menopause, a large proportion of women experience centralized weight gain and increased risk of chronic diseases associated with insulin resistance, dyslipidemia and hypertension. Thus, we will enroll women who are overweight/obese in this pilot study.

PRELIMINARY DATA AND INVESTIGATIVE TEAM EXPERIENCE

We do not yet have any of our own preliminary data on laboratory sitting studies so our protocol is modeled after two published studies.[12, 13] Dr. Sears has recently completed a randomized controlled trial of a diet intervention for obese participants with the Metabolic Syndrome. Most of the clinical procedures and assessments that we propose to conduct in the current proposal were elements of her Diet Study (UCSD IRB#110549). These include recruitment of appropriate participants, coordination with the UCSD Clinical and Translational Research Institute (CTRI) Center for Clinical Research (CCR) clinic staff, blood and urine sample collections, and meal tolerance tests (**Figure 1**). Most of the blood and urine analyses proposed herein were used by Dr. Sears in her Diet Study protocol including measures of insulin, glucose, and targeted mitochondrial metabolites. Dr. Schenk (Co-investigator) has clinical experience with meal tolerance tests and is an expert in exercise physiology. Dr. Galina Khemlina, M.D., who also was a member of Dr. Sears' Diet Study team and whose clinical specialty is internal medicine - geriatrics, will assist with blood test orders and general clinical oversight. Graduate student Michelle Black and Katie Crist from the Department of Family Medicine and Public Health Joint Doctoral Program in Public Health will assist the team with study coordination, recruitment, and biospecimen processing.

Figure 1. Meal tolerance test glucose excursion data from Sears Diet Study, before and after diet intervention (manuscript in preparation). N=16. *p*-value <0.05 for intervention-induced change in glucose excursion by MANOVA repeated measures.

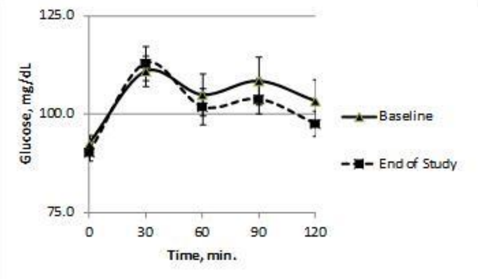

Studies of Sedentary Behavior. Drs. Sears and Kerr and collaborators have completed and on-going sedentary behavior studies in older adults. Kerr, et al., recently published a two-arm randomized pilot intervention to decrease sitting time and increase sit-to-stand transitions in working and non-working older adults.[32] In these studies, participant wore ActivPAL devices for 21 consecutive days while their "real-world" behaviors were measured. Participants achieved a 2-hr increase in standing per day and average standing bouts were 10-15 min each. This published pilot study demonstrates that this length of standing break is feasible even in people who sit for ≥8 hr/day. Drs. Kerr and Sears have recently published a study on gender and age differences in hourly and daily patterns of sedentary time in older adults living in retirement communities.[33]

8. PROGRESS REPORT

N/A

9. RESEARCH DESIGN AND METHODS

Overview. We will enroll 10 sedentary, overweight/obese postmenopausal women 55+ years of age as

described above. Participants will visit the clinic on 4 occasions for a 6-hour monitoring period and each time follow one of the 4 sitting protocols, one protocol per visit, in random order following a one-hour sitting run-in period: (Protocol A) 5 hours sitting; (Protocol B) 5 hours sitting including 15 2-minute standing interruptions, 3 per hour; (Protocol C) 5 hours sitting including 5 2-minute walking interruptions, 1 per hour; and (Protocol D) 5 hours sitting including 5 10-minute standing breaks. All study protocols include baseline, mid-point, and end-of-study bathroom breaks for urine sample collection with additional bathroom breaks allowed as needed. See **Figure 2** below for schematic of protocols. Controlled sitting interruption interventions varying in interruption length and type will allow us to gauge their impact on metabolic, mitochondrial and endothelial functioning during each protocol and gauge their probable impact on chronic health outcomes. We will collect blood pressure and femoral flow-mediated dilation (FMD) measurements, and blood and urine to assess changes in biomarkers of mitochondrial function and meal tolerance (glucose and insulin areas under the curve) over the course of the sitting protocols. Our study is a crossover design and each participant will be her own control. **We hypothesize that frequency of sit-to-stands during a 5-hour sitting period will result in health benefits that can be observed with a simple 2-minute standing interruption, and that this will be associated with improvements in metabolism and endothelial function**.

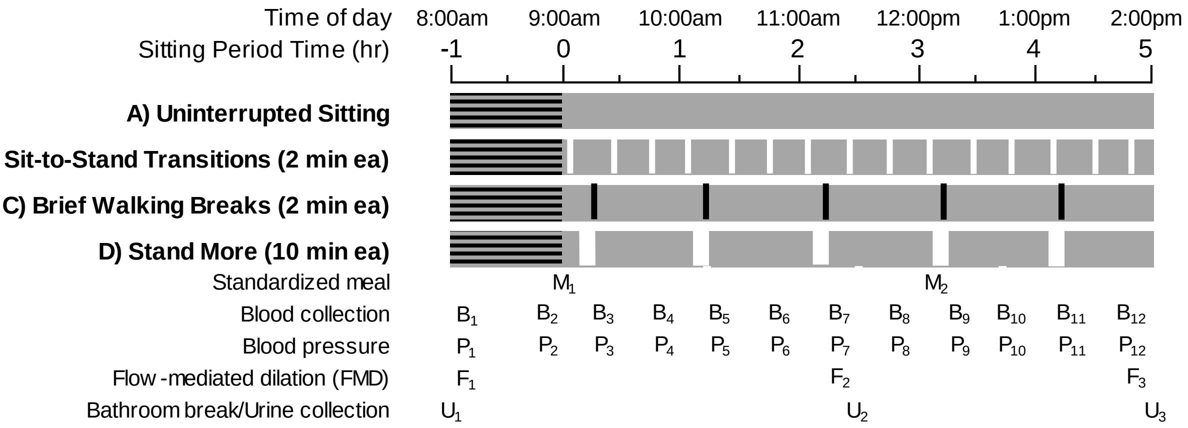

Figure 2. Schematic of sitting protocols and biospecimen/clinical monitoring. Participants will be assigned to complete the four protocols (A-D) in random order. MTT – Meal Tolerance Test of an Ensure Plus® mixed meal shake.

Rationale and expected outcomes. We hypothesize that engagement of muscles and increased blood flow to extremities associated with sit-to-stand transitions are key beneficial aspects of frequent sit-to-stand interruptions of sitting time that are not realized by simply reducing total sitting time. Protocol B will include frequent sit-to-stand transitions (15 total) and reduce sitting across the 5-hr period by 10%. In comparison, Protocols C & D will include less frequent sit-to-stand transitions (5 total each) and will reduce total sitting time across the 5-hr period by 3.3% and 16.7%, respectively. We speculate that repeated, frequent sit-to-stand transitions more-effectively activate muscle mitochondrial metabolic pathways and enhance endothelial function, facilitating nutrient processing, reducing oxidative stress and preserving insulin action, across the day. We can assess acute activation of these pathways by measuring plasma mitochondrial metabolites (lipids, amino acids), and blood pressure and FMD, comparing the change in these during our four sitting protocols. We will gauge sitting protocol effects on insulin sensitivity using two sequential liquid meal tolerance tests per visit to measure postprandial excursion of glucose and insulin (area under the curve, AUC), as we have done previously (**Figure 1**) and as others have done in laboratory sitting studies.[12,13]

We will study postmenopausal women in this pilot study and in the program project grant proposal that we are resubmitting this May 2016. Postmenopausal women are the fastest growing age/sex population subset in the

United States. Postmenopausal women experience a high prevalence of metabolic disturbances; prior to, during, and post-menopause, a large proportion of women experience centralized weight gain and increased risk of chronic diseases associated with insulin resistance, dyslipidemia and hypertension. Aging is associated with increase sedentary behavior and postmenopausal women make up the majority of the U.S. population over the age of 65 years. Thus, this at-risk population is highly appropriate for our sitting interruption studies.

Expected Outcomes

We expect that sitting interruptions (Protocols B-D above) will lead to improved metabolic profiles over the 5hr sitting time as compared to 5hr of nearly-continuous sitting (Protocol A above); these will be associated with commensurate improvements in insulin and glucose AUC, change in mitochondrial biomarkers, and improvements in blood pressure and FMD over the 5-hr period.

- We speculate that simple frequent sit-to-stand transitions (Protocol B) will be as impactful as the brief walking breaks condition (Protocol C) and the standing more condition (Protocol D), compared to the control uninterrupted sitting condition (Protocol A).
- A practical, behavioral intervention for increasing sitting interruptions and improving healthy aging outcomes is an expected outcome of the project.

Research Methods. All procedures and laboratory analyses will be conducted at UCSD.

Participant screening. Participants will initially be screened for eligibility by phone to establish postmenopausal status, age, fluency in English, inclusion BMI, and absence of exclusionary sedentary time, medical or weight change criteria (see below). Initially eligible participants will be scheduled for the CCR screening visit and sent the consent, HIPAA, and Subject Bill of Rights forms to review before the screening visit. At the screening visit, participants will be thoroughly informed about the study and, if willing, consented. 12mL of blood will be drawn for measure of baseline biomarkers, complete blood count (CBC, to measure hemoglobin levels), and assessment of glucose and diabetes control (hemoglobin A1c); vitals, height, weight will be collected. Participants will complete a general health questionnaire with an anonymized identifier. No protected health information will be included on the questionnaire. We will conduct the Short Physical Performance Battery tests to asses physical functioning in participants and to confirm that participants can walk, stand without falling, and perform a chair rise. Participants will be given a granola bar-type snack before they leave the CCR clinic.

Eligibility of participants regarding sedentary behavior criteria will be determined by self-report.

Eligible participants will be invited to participate in the full study and scheduled for 4 sitting protocol clinic visits with 7-10 days between each visit. Enrolled participants will be given 4 market-variety, prepared, meals from the study investigators for their consumption the night before each sitting protocol visit. Study investigators will communicate with participants by email or regular mail, as preferred by each participant, to send out consent, HIPAA, and Bill of Rights forms and directions to the CCR clinic prior to the Screening Visit, to coordinate scheduling of clinic visits, and to send appointment reminders.

Sitting protocol visits. Participants will consume the same study-provided meal the night before each clinic visit, abstain from alcohol consumption and moderate-to-vigorous exercise 48 hrs before each clinic visit, and fast for at least 10 hrs before arriving in the morning for each clinic visit. Participants will be asked to drink a glass of water during or just prior to their travel to the clinic (to facilitate initial urine collection) and to wear comfortable clothing including shorts, a dress or a skirt (to facilitate FMD studies). After arriving at the clinic, participants will be outfitted with a small I.V. catheter, and an automatic blood pressure monitor (ABPM). Blood pressure (BP) and FMD measurements, 10mL blood, and ~15mL urine will be collected at the start of a 1-hr lead-in sitting time. BP and blood samples will be collected every 30min thereafter, as designated in **Figure 2** above, with only 3mL blood collected at each interim sampling until a final 10mL blood draw. Additional urine samples will be collected at 2.5hr and 5hr of the sitting period. An additional FMD measurement will be collected at 5hr of the

sitting period. **Figure 2** shows that timing of all study events. Participants will drink a mixed meal shake (Ensure Plus®, 5 kcal/kg body weight) within 5min time at both the 0hr and 3hr sitting period time points. All biospecimen, blood pressure and FMD sampling and participant sitting interruption activities will be time-stamped. At the end of each visit, participants will complete a brief survey about their perceived energy level and protocol burden, each on scale of 1 to 5. In addition, at the end of their first study visit, we will ask about their potential willingness to participate in muscle biopsies. If the response to biopsies is positive in our participants, we may consider including biopsies in future sedentary behavior studies.

Participants will be allowed non-protocol bathroom breaks, as needed, the times of which will be recorded. All bathroom breaks/urine collections will occur in a restroom <20 feet from the study room chair. Participants will be seated in a comfortably cushioned but firm, straight-back arm-chair and will be allowed to use their arm strength to assist themselves with standing, if needed. All biospecimen collection time points: Plasma and buffy coats will be isolated from whole blood collected in EDTA-containing tubes. Plasma, buffy coats, and urine will be frozen and stored at -80°C for future analyses. Participants will be allowed to drink moderate amounts of water throughout the protocols and given a granola bar-type snack at the end of each day before they leave the CCR clinic facility. The study protocol includes a 6-hour monitoring period (1-hour run-in followed by 5 hours sitting ± standing or walking interruptions, all including a mid-point bathroom break for urine collection). We anticipate that the total visit time will be 7 hours, as it will take ~30min each for set-up activities before (setup of the devices, placement of the I.V. catheter, baseline urine collection) and wrap-up activities after (removal of devices and I.V. catheter, end-point urine collection, provision of snack) the 6-hour monitoring period.

Sitting protocol visits will be scheduled with 7-10 days between each visit. Total time span from the screening visit blood draw through the 4th sitting protocol visit will be a minimum of 35 days and a maximum of approximately 44 days, roughly 5-6 weeks.

Participants will be allowed to take their oral prescription medications at their usual time of day with the following caveats: 1) if they take them in the morning with water, they can take them before arriving at the clinic; 2) if they need to take them with food, they can bring them to the study clinic and take them after their snack (screening visit) or after their breakfast meal (study visits 1-4); 3) if they take vasodilator medications (e.g., nitrates, ACE inhibitors) before 2:00pm each day, they will be asked to delay their dose until after the last study measurement at 2:00pm. They will be asked to bring their medications with them and take them before leaving the CCR clinic. For ANY participant who has a study visit blood pressure measurement greater than our screening limit (SBP ≥165 or DBP ≥100), we will terminate the study-portion activities of the visit and have vasodilator-taking participants take their medication immediately.

Biospecimen analyses. Blood will be collected (55 mL total per day) in EDTA tubes and processed by centrifugation for plasma aliquotting. Urine (15mL x 3 collections) will be centrifuged to remove cell debris and aliquotted. See **Figure 2** for schematic of biospecimen collections during protocols. Whole blood, plasma and urine samples will be stored immediately at -80°C in tubes labeled with de-identified participant study ID. Urine samples will be stored for possible ancillary studies of oxidative stress biomarkers that are beyond the scope of the current proposal. All assessments and assays described below are familiar to our investigative team from previous work including glucose and hormonal analyses and metabolomics. Dr. Sears will store de-identified biospecimens in her laboratory freezers in the Stein Clinical Research Building.

Metabolic parameters. Fasting and postprandial glucose and insulin concentrations will be determined using traditional assays. The meal tolerance tests (using Ensure Plus® shakes, as in **Figure 1**) will include blood sampling as noted in **Figure 2** to measure postprandial blood glucose, insulin, and mitochondrial metabolites, which we will use to assess insulin sensitivity and mitochondrial functioning. Targeted metabolomic analysis of fasting and postprandial plasma will be conducted at the UCSD Biochemical Genetics and Metabolomics

Laboratory (Lead – Dr. Bruce Barshop).

Endothelial function. Endothelial function will be indirectly assessed by ambulatory, automatic blood pressure monitoring every 30min. We will save plasma samples for possible ancillary studies of endothelial/vascular injury and inflammation biomarkers CRP, sICAM-1, cVCAM-1, SAA measurements in samples collected at baseline and at the end of each visit protocol using standard immuno-based methods.

Endothelial function will be directly assessed by femoral flow-mediated dilation (FMD) at baseline, mid-point and at the end of each visit (see **Figure 1**). The FMD procedure will be conducted by Minaxi Trivedi, RDCS, RDMS, RVT, who is an ultrasound technical expert with 25 years' experience and a member of the CTRI CCR staff resource team. She conducts FMD for clinical studies conducted at the CCR. Briefly, the FMD protocol is as follows and is based on similar published studies [24,26,27]: 1) place ECG leads; 2) using an ultrasound probe, scan the superficial femoral artery and select the location with clear anterior and posterior intimal interfaces; 3) establish landmarks on the thigh to make sure same area is being scanned in same-day measures and mark the skin with a felt-tip pen; 4) set instrument for a 5 heart beat cycle; 5) obtain pulse Doppler velocity signal from a mid-artery sample of images; 6) acquire ultrasound images (Baseline); 7) place blood pressure cuff distal to scan area; 8) elevate cuff pressure to 250 mmHg systolic BP; 9) leave cuff inflated for 5 minutes then immediately deflate cuff and start imaging of femoral artery; 10) continuously record longitudinal images of the femoral artery from 30 seconds prior to 5 minutes after cuff deflation; 11) obtain pulsed Doppler signal immediately upon cuff release and no later than 15 seconds after cuff deflation to assess hyperemic velocity; 12) annotate on the screen Angle (degree), Distance (cm), CI (cuff inflation time), and CD (cuff deflation time); 13) finish acquiring ultrasound of femoral artery images in 5 minutes. The cuff and ECG lead wire are then removed from the participant until the next measurement in the sitting protocol. Dr. Luis Castellanos, MD, will conduct the reading and quantitation of the FMD data generated by Ms. Trivedi, as he has done for his own CTRI CCR-based FMD studies.

Statistics**.** Although this is a pilot study, we will benefit tremendously in assessing the practicality and efficacy of our 4 sitting protocols. We may not have sufficient statistical power to detect significant differences induced by the sitting interruption protocols in our 10 participants. However, based on published laboratory sitting studies[12,13,24] and our own clinical MTT studies (Sears Diet Study manuscript in preparation and **Figure 1**), we anticipate being able to detect change trends in the parameters we propose to measure. Briefly, we will use MANOVA repeated measures with Tukey's post hoc test to evaluate changes in metabolic parameters over time and between sitting protocol arms per participant. P-values less than 0.05 will be considered significant. Multivariate regression and principal components analysis will be used to assess associations between metabolic and clinical biomarker parameters and sitting protocol type.

Limitations and alternatives. We may have challenges enrolling participants for four 7-hour clinic visits. If this is the case, we will restrict our enrollment to participants who do not work full time. The UCSD CTRI clinic is available to expand their hours to include Saturday if there is significant need so this may help us enroll participants with full-time employment and who can only come to clinic on a Saturday.

10. HUMAN PARTICIPANTS

We will enroll 10 postmenopausal, female participants in this pilot study. The inclusion/exclusion criteria for potential participants are as follows:

Inclusion criteria:

1) Postmenopausal woman, any ethnicity/race, 55+ years of age.
2) Ambulatory, medically stable, able to give informed consent, and safely complete the protocols.
3) Fluent in the English language.

4) Body Mass Index range of 18-45kg/m2.
5) Sedentary: Average ≥6hr sitting time, and ≤20min physical activity on less than 3 days per week as assessed by self-report

Prisoners will not be used for this study.

Exclusion criteria include:

1) Unable to complete the Short Physical Performance Battery.
2) Mental states that would preclude complete understanding of the protocol and compliance.
3) Chronic illness that may be associated with weight change (HIV/AIDS, active cancer, or uncontrolled thyroid disease).
4) Body Mass Index <18 or >45kg/m2.
5) Anemia (hemoglobin ≤11g/dL) – determined by CBC test done at screening.
6) Arthritis or degenerative joint disease affecting knees where repeated sitting/standing interruptions might cause pain.
7) Personal or family history of venous thrombosis.
8) Type 1 diabetes mellitus
9) Poorly controlled hypertension (SBP ≥165 or DBP ≥100).
10) Weight instability in past 3 months (no more than 5% up or down).
11) Uncontrolled diabetes.[34,35] Defined in participants with fasting glucose >130mg/dL on 2 separate capillary readings taken over 2wks time if diabetes medications have been changed in prior 3 months. If no medications or no recent medications changes, defined as participants <65 years of age with HbA1c ≥53 mmol/mol (7%) and participants ≥65 years of age with HbA1c ≥58mmol/mol (7.5%).
12) Use of insulin medications.
13) Regular use of vasodilator medication AND high risk of stroke and/or heart attack (i.e., history of multiple hospitalizations (>2X in the last 6 months), congestive heart failure, atrial fibrillation, and/or stroke).
14) Use of any immunosuppressant or corticosteroid medication.
15) Use of medications that might cause weight change (e.g., second generation anti-psychotics).
16) < 6hr average daily sitting time.
17) >20min physical activity average on 3 or more days per week
18) Participating in another clinical trial.
19) Blood donation less than 56 days prior to screening visit.
20) Smoking cigarettes or anything, and other use of tobacco products.

There are no collaborating sites for participant recruitment or enrollment.

11. RECRUITMENT AND PROCEDURES PREPARATORY TO RESEARCH

Participants will be recruited from the community and UCSD campus and clinics by approved and posted flyers, Craig’s List postings (using approved text), from ResearchMatch, and from lists of potential participants who have freely expressed interest in research studies. The text of all recruitment materials will be reviewed and approved by UCSD HRPP. We have a recruitment flyer and matching Craig’s List text at this time. Additional flyers, if needed, will be sent to IRB for approval. Recruitment efforts will include the use of ResearchMatch, which is a NIH-sponsored national registry of volunteers who have indicated a willingness to learn more about research studies. Dr. Kerr is registered with the ResearchMatch registry. ResearchMatch sends a recruitment message to potential participants who meet the criteria specified. Interested participants will then be contacted by study staff to determine eligibility. We have submitted our ResearchMatch letter for review. Potential participants will be directed to call Ms. Crist or CTRI staff and will be pre-screened for likely eligibility over the phone under a waiver of written consent by her or one of the other co-investigators. A script for the oral consent and pre-screening questions to determine likely eligibility has been submitted for IRB review. Drs. Sears and Kerr have a list of potential participants who have freely expressed interest in research studies which they will use recruit

participants in this study. Eligible participants will be asked for their UCSD medical record number or be asked to call 888-309-8273 (option 2) to register for a UCSD medical records number.

Exclusion of vulnerable participants
Compensation provided is not high enough for economic incentive, in our opinion. Ample time will be provided to give information and answer questions from potential participants, and phone follow-ups will be encouraged if the person has further questions. Potential study participants must be able to provide informed consent; thus, we do not anticipate enrolling mentally ill or educationally disadvantaged. We will ensure that any potential participant including students, postdoctoral fellows, or UCSD/VASDHS employees knows that they have the right to refuse enrollment in our study. This is clearly written in our consent form.

12. INFORMED CONSENT

Informed consent will be obtained before any person can participate in the study. Study investigators (all research team members listed on section 21) will obtain informed consent in the private setting of the CTRI. The contents and process of obtaining informed consent will be in accordance with all applicable regulatory requirements. Potential participants will be pre-screened for likely eligibility over the phone under a waiver of written consent. The investigators will fully explain to the participant the purpose of the study and all of its procedures, as well as its potential risks prior to entering into the study. A copy of the consent form(s) has been electronically attached to this application. All participants are recruited in collaboration with a study investigator in a private setting. The study will be described in detail by the study investigator in a private setting at the CTRI. Signed consent forms will always be approved by Dr. Dorothy Sears. As part of the consent language, participants will be informed that UCSD IRB will have access to their participant files. Participants will be informed that they may withdraw from the study at any time. There are no safety issues with early withdrawal. All information generated in this study and all study documents related to this study must be considered as highly confidential and must not be disclosed to any persons not directly involved with the study without written permission from the principal investigator; only an acronym and numbers will be used to identify participants in the database. The code for enrollment begins with the number 001 and continues in ascending sequential order. The UCSD IRB and the Research and Development Committee must approve the informed consent form and all protocol amendments. The IRB requires that information given to the participant as part of the participant informed consent is in accordance with 21 CFR 50.25 and that the Board approves it. Any major modifications to the protocol including change in study drug, change in body fluid sampling or change in P.I. must first be approved by the UCSD IRB. All participants will be informed of changes to the protocol. Any minor changes to the protocol will be submitted to all oversight bodies named above with the annual report. If deviations from the protocol are made by investigators, other study personnel or participants, we will notify the UCSD IRB within 10 days unless participant safety is compromised; in the latter case, reports will be submitted within 48 hours of knowledge of the protocol deviation.

Eligibility screening by phone: Potential participants will have the study purpose and procedures described to them during an initial eligibility screening that make take place over the phone or in-person by CITI certified and trained research or CTRI staff member. We will obtain and document verbal consent to participate in the eligibility screening. We are requesting a waiver of written consent for the telephone screening of potential participants because the telephone screening presents no more than minimal risk of harm to participants and involves no procedures for which written consent is normally required outside of the research context.

Consenting and eligibility screening in person: If eligible, participants will be mailed the consent form, HIPAA authorization and copies of the Experimental Participants Bill of Rights. Consent forms, HIPPA authorization forms, and Experimental Participants Bill of Rights will be provided in English; lack of proficiency in English is an exclusion criterion for this study. CITI-certified and trained study staff (recruitment and measurement staff) will schedule a follow up phone call with the potential study participant to review and answer questions

about the forms. The potential participant may take as much time as needed to understand the study and ask questions. After reviewing the consent form and speaking with study staff, participants who want to participate will be scheduled for their screening clinic visit during which time they will present their signed consent, Participants Bill of Rights, and HIPAA forms to study investigators before any study related procedures are performed. A copy of the signed forms will be given to the study participant.

Study Enrollment: Participants will be enrolled if they meet the behavioral inclusion criteria, including sedentary behavior of ≥6 hours of sitting, , and 20min physical activity on less than 3 days per week. Participants that meet these criteria will be invited to enroll in the full study and scheduled for their 4 study clinic visits.

A waiver of documented consent is being requested to enable us to screen participants over the phone. Specifically, participants will be recruited from the community using recruitment flyers. Interested persons will be asked to call a study staff member for consideration of participation in this study. When they call, the study staff member will screen them over the phone by asking them questions to ensure that they meet all of the inclusion criteria and none of the exclusion criteria. A phone script has been uploaded to eIRB that describes this interaction on behalf of the study staff member. If the person meets all the study criteria, they will be scheduled for a screening visit at which time their eligibility will be verified. After the participant has signed the informed consent form, the screening document will be attached to the signed consent form and filed in secured premises.

We are requesting a waiver of documented consent for the screening as this part of research presents no more than minimal risk of harm to participants and involves no procedures for which written consent is normally required outside of the research consent.

13. ALTERNATIVES TO STUDY PARTICIPATION

The alternative to participating in this study is to choose not to participate. There are many publicly available resources for learning about how to reduce sedentary behavior.

14. POTENTIAL RISKS

Potential risks to this study are minimal. Participants may experience 1) anxiety or embarrassment related to answering questions about one's health or to the measurement process; 2) concern for privacy related to divulging personal information on the survey or loss of confidentiality if computer is accessed by an unauthorized person; 3) increased knowledge of sedentary behavior may provide more information about participants' habits and behaviors that they may wish to keep confidential; 4) discomfort from wearing any of the devices during the study clinic visits; 5) pain related to venipuncture; 6) discomfort and /or boredom during prolonged sitting (up to 6hrs including 2 bathroom breaks per protocol, **Figure 2**, and additional bathroom breaks allowed as needed); 7) discomfort performing standing interruptions repeatedly (up to 3X per hour for 5 hours); 8) deep vein thrombosis (during the control protocol, participants will sit for 2 back-to-back intervals of uninterrupted sitting (3.5 hours and 2.5 hours) with a standing bathroom break/urine collection in-between, see **Figure 2**); 9) anemia due to withdrawal of 232mL of blood over 5-6 weeks (55mL maximum per study visit); 10) extremely high blood pressure (increasing risk for stroke and heart attack) for those participants withholding morning vasodilator medication dose until study measurement completion at 2:00pm.

Judging from our past experience and the current literature, these risks are relatively slight and of low likelihood.

15. RISK MANAGEMENT PROCEDURES AND ADEQUACY OF RESOURCES

Risks will be minimized by the following procedures:

1) Risks of embarrassment or social discomfort will be minimized by fully informing participants of the topics to be discussed and the specific involvement required of them before they agree to participate. Participants will be informed that they may discontinue their involvement at any time, with no impact on the incentives previously received.

2) Participants will be told that all responses are confidential. Risks to confidentiality will be minimized by keeping informed consent statements and participant data in separate locked file cabinets so that individuals are not easily connected to the study results. All sensor data will be stored on a firewall and password-protected project server at the UC San Diego. To reduce the risk to (and fear of) confidentiality, individual identifiers will be stripped from all participant records and data following data collection. We will assign each participant a study ID and all records will be coded with the study ID rather than personal identifiers. The code that links the study ID and the name will be stored in a separate place than the data file until all of the measurements are complete. At that point, the personal information will be destroyed and all analyses will be conducted with the data set that has no personal identifiers. No identifying data will be collected in the field, only in a secure clinical research clinic. All data will be kept in locked cabinets at the study office, accessible only by investigators and project staff. Participants will also be told about the confidentiality procedures and that they have the right to refuse to answer questions or to terminate their participation in the study at any time without prejudice. The PI will assure that all human protections standards are met.

Participants will be told that the UCSD Institutional Review Board will have access to research records.

3) The blood pressure cuffs (for BP and FMD measurements) will be adjusted to be as comfortable as possible while maintaining functionality. Discomfort with this is rare, in our experience.

4) Venipuncture will be performed by trained and certified phlebotomists that are members of the UCSD CTRI CCR staff. The risks of drawing blood include temporary discomfort from the needle in the participant's arm, bruising, swelling at the needle site, and, in rare instances, infection or feeling faint. Care will be taken to minimize these risks and use of aseptic techniques will minimize the risks associated with venipuncture.

5) Sitting discomfort will be minimized by the participants' sitting in a comfortably cushioned but firm, straight-back arm-chair. In order to minimize boredom, participants will be permitted to read, play games, and/or watch DVDs/TV during their extended sitting time.

6) Standing discomfort from performing standing interruptions repeatedly (3X per hour for 5 hours in Protocol B) will be minimized by screening for knee arthritis/disease and by allowing participants to stand up at their own speed. Participants will be allowed to use their arm strength to facilitate standing. We have added the Short Physical Performance Battery test at screening to assess participants' ability to safely and comfortably perform the sitting interruption protocols.

7) We will minimize risk of study-related deep vein thrombosis during the control protocol by including a bathroom break/urine collection in-between back-to-back 3.5-hour and 2.5-hour intervals of uninterrupted sitting (see **Figure 2**) and by excluding women who have a personal or family history of thrombosis.

8) Twelve mL of blood will be removed at screening. At each of the four subsequent study visits, 55mL of blood will be removed during the 6-hour protocols. Over the entire 5-6 week study period, 232mL of blood will be removed. If a participant's weight is less than 110lbs, a maximum of 50mL of blood will be removed during the 6-hour protocols. Participants must not have donated blood less than 56 days prior to screening, will be advised not to donate blood during the study, and advised to wait to donate blood for 56 days after completing the study.

9) We have added an exclusion for people who take vasodilator medication AND who have a high risk of stroke and/or heart attack (i.e., history of multiple hospitalizations (>2X in the last 6 months), congestive heart failure, atrial fibrillation, and/or stroke). For ANY participant who has a study visit blood pressure measurement greater than our screening limit (SBP ≥165 or DBP ≥100), we will terminate the protocol-portion activities of the visit and have vasodilator-taking participants take their medication immediately.

The CTRI facilities and the resources of Dr. Dorothy Sears and her team are adequate for conducting this study and assure the protection of study participants, timely completion of the study protocols, and sample analyses.

Exclusion of vulnerable participants

Compensation provided is not high enough for economic incentive, in our opinion. Ample time will be provided to give information and answer questions from potential participants, and phone follow-ups will be encouraged if any participant has further questions. Potential study participants must be able to provide informed consent; thus, we do not anticipate enrolling mentally ill or educationally disadvantaged. We will ensure that any potential participant including students, postdoctoral fellows, or UCSD/VASDHS employees knows that they have the right to refuse enrollment in our study and are under no pressure or risk of repercussion and that concerted efforts will be made to keep their personalized data private. Statements to these effects are clearly written in our consent form. We are only enrolling postmenopausal women, thus, pregnancy is not a concern for our study population.

Data and Safety Monitoring Plan

Drs. Dorothy Sears, Jacqueline Kerr, Victor Legner, Galina Khemlina, and Simon Schenk will be responsible for the data safety and monitoring plan. All adverse events (side effects) will be captured during the study visits and by phone, as needed. Drs. Legner and Khemlina will assess and assign the severity and relationship of all reported adverse events. All events will be documented in the participants' charts. In the event of a serious adverse event the investigator will notify the Institutional Review Board within 10 days of the occurrence and provide a detailed write-up and all the relevant information needed to document the case. Complications that arise from the study outside of the standard-of-care follow-up will be cared for by UCSD.

16. PRIVACY AND CONFIDENTIALITY CONSIDERATIONS INCLUDING DATA ACCESS AND MANAGEMENT

We have established an extensive protocol to protect participants' privacy. We will keep informed consent statements and participant data in separate locked files cabinets so that individuals are not easily connected to the study results. All sensor data will be stored on a firewall and password-protected project server at the UC San Diego. All participant records and data will be stripped of individual identifiers following data collection. We will assign each person a study ID and all records will be coded with the study ID rather than personal identifiers. The code that links the study ID and the name will be stored in a separate place than the data file until all of the measurements are complete. At that point, the personal information will be destroyed and all analyses will be conducted with the data set that has no personal identifiers. All data will be kept in locked cabinets at the study office, accessible only by investigators and project staff. Dr. Sears will store de-identified biospecimens in her laboratory freezers in the Stein Clinical Research Building. Participants will also be told about the confidentiality procedures and that they have the right to refuse to answer questions or to terminate their participation in the study at any time without prejudice. Finally, participant data will not be sold or exchanged with anyone and data sharing as per NIH requirements will be performed within strict protocol-driven procedural guidelines. The PI will assure that all human protections standards are met.

The principal investigators will review any confidentiality breeches on a case-by-case basis to determine whether study procedures should be modified to prevent recurrence. We will inform participants about the potential for confidentiality breeches through informed consent documents. Throughout the study periodic checks will take place to make sure that participants' data is coded correctly, that all files have been password protected, and that privacy information is kept in a secure location. Should an issue arise, the PI will notify all staff working on the

project and update the policies and procedures with the IRB as needed, and only upon IRB approval will implementation take place.

The study investigators do not reasonably expect to collect any information that Federal, State, and/or local laws/regulations require to be reported. Study participants are informed of this as outlined in the consent HIPAA form.

17. POTENTIAL BENEFITS

Participants may or may not receive any benefit from participating in the study. They may have an increased awareness of their sedentary behavior.

Participants will contribute to science to improve methods of assessing the mechanisms by which sedentary behavior contributes to chronic disease risk and how this risk might be mitigated by interruption of extended sitting bouts. Independent of physical activity (i.e., exercise) sedentary behavior is known to be associated with increased risk for cancer, type 2 diabetes, cardiovascular disease and all-cause mortality. This study will broaden our understanding of the impact of sedentary behavior on these risks. Results will inform public health policies and guidelines, and that could have an important impact on populations on a relatively permanent basis. Any information that might be used to reduce sedentary behavior could have important implications for preventing or delaying the development of type 2 diabetes, cancers, cardiovascular disease and other morbidities. The value of information gained as a result of this study is highly likely to outweigh any minimal risks or minor inconveniences to the individuals who participate in this research.

18. RISK/BENEFIT RATIO

The minor potential risks of participating in this study are outweighed by the potential benefits of improved awareness about sedentary behavior by the potential benefit to the research field. Therefore, the risk-to-benefit ratio is reasonably low for the study participants and for society in general.

19. EXPENSE TO PARTICIPANT

Travel to the CTRI CCR for the screening and study visits is the only expense that we can foresee for the participants and this will be offset by the per visit compensation.

20. COMPENSATION FOR PARTICIPATION

Travel compensation of $10 will be provided for the initial Screening Visit and if participants are eligible to proceed to the behavior screening. Enrolled participants can receive up to $410.00 total compensation for their time and travel associated with participating in this study. If a participant does not complete the entire study, their payment will be pro-rated given at the completion of the following: $10.00 for Screening Visit, $50.00 for Visit 1, $50.00 for Visit 2, $50.00 for Visit 3, and $250 for Visit 4. Participants will also be provided with the study dinner meals to be eaten the night before each of the 4 protocol clinic visits and a granola bar-type snack at the end of each of the Screening and Study Visits.

21. PRIVILEGES/CERTIFICATIONS/LICENSES AND RESEARCH TEAM RESPONSIBILITIES

All of the study co-investigators have completed the appropriate CITI training. Drs. Legner and Khemlina have privileges at the UC San Diego Health System and the VA San Diego Healthcare System and are certified to perform the procedures outlined in this protocol at UCSD. They are both specialists in internal medicine and geriatrics.

The procedures to be performed by the physician Co-Investigators are:

- To review and assist in the study protocol design.
- To review clinical laboratory tests, including the measurement of FMD, HbA1c and CBC, and blood pressure.
- To provide clinical oversight of study protocol and possible adverse events.

The following research team members have UCSD appropriate medical privileges:

- Victor Legner, M.D., M.A., UCSD Associate Clinical Professor of Medicine
- Galina Khemlina, M.D., UCSD Assistant Clinical Professor of Medicine
- Luis Castellanos, M.D., M.P.H., UCSD Assistant Clinical Professor of Medicine

The following members will be in charge of telephone screening and scheduling participants, entering study related documents, filing documents and submitting all paperwork to the IRB, and executing the protocol during the participants' study clinic visits.

- Dorothy Sears, Ph.D., UCSD Associate Professor of Medicine
- Simon Schenk, Ph.D., UCSD Assistant Professor of Orthopedic Surgery
- Michelle Black, UCSD Graduate Student
- Katie Crist, UCSD Program Manager
- Lindsay Dillon, UCSD, Project Coordinator
- Daniela Vital, UCSD, Student Intern
- Basma Abdellaoui, UCSD, Student Intern

The following non-physician research team members will be involved in obtaining informed consent and health history from participants at the start of and during the screening clinic visit:

- Michelle Black, Graduate Student
- Katie Crist, UCSD Program Manager
- Lindsay Dillon, UCSD, Project Coordinator
- Cynthia Knott, R.D., CTRI, CCR Staff member
- Simon Schenk, Ph.D., UCSD Assistant Professor of Orthopedic Surgery
- Daniela Vital, UCSD, Student Intern
- Basma Abdellaoui, UCSD, Student Intern

The following non-physician research team member will be involved in conducting femoral flow-mediated dilation (FMD) assessments during the 4 study visits:

- Minaxi Trivedi, R.D.C.S., R.D.M.S., R.V.T., CTRI CCR Staff member

Dorothy Sears (PI) will be in charge of overseeing all financial aspects of the study (i.e. budget, CTRI costs and payments, participant reimbursement, etc.) with the assistance of Julie Wilkes, her UCSD Department of Medicine Fund Manager. Dr. Sears will ensure that every participant reads and signs the informed consent form prior to any screening procedures being performed;

The following investigators will be responsible for data analyses, statistical tests, interpretation of results, and manuscript preparation, if appropriate.

- Dorothy Sears, Ph.D., UCSD Associate Professor of Medicine
- Simon Schenk, Ph.D., UCSD Assistant Professor of Orthopedic Surgery
- Michelle Black, UCSD Graduate Student
- Jacqueline Kerr, Ph.D., UCSD Associate Professor of Family Medicine & Public Health
- Loki Natarajan, Ph.D., UCSD Associate Professor of Family Medicine & Public Health
- Victor Legner, M.D., UCSD Associate Professor of Medicine
- Galina Khemlina, M.D., UCSD Assistant Clinical Professor of Medicine
- Luis Castellanos, M.D., M.P.H., UCSD Assistant Clinical Professor of Medicine
- Bruce Barshop, M.D., Ph.D., UCSD Professor of Pediatrics
- Basma Abdellaoui, UCSD, Student Intern

Members of the research team performing tissue and blood analyses on samples that contain only research codes include:

- Bruce Barshop, M.D., Ph.D., UCSD Professor of Pediatrics
- David Boyle, B.S., UCSD Professor of Medicine
- Simon Schenk, Ph.D., UCSD Assistant Professor of Orthopedic Surgery
- Dorothy Sears, Ph.D., UCSD Associate Professor of Medicine
- Basma Abdellaoui, UCSD, Student Intern

Support for study involvement:

- Supported by funds from the Department of Family Medicine and Public Health Women's Health Center of Excellence:
  Dorothy Sears, Ph.D., UCSD Associate Professor of Medicine
  Michelle Black, UCSD Graduate Student

22. BIBLIOGRAPHY

1. Saunders TJ, Tremblay MS, Mathieu ME, et al. Associations of sedentary behavior, sedentary bouts and breaks in sedentary time with cardiometabolic risk in children with a family history of obesity. *PLoS One.* 2013;8(11):e79143.

2. Helmink JH, Kremers SP, van Brussel-Visser FN, de Vries NK. Sitting time and Body Mass Index in diabetics and pre-diabetics willing to participate in a lifestyle intervention. *Int J Environ Res Public Health.* Sep 2011;8(9):3747-3758.

3. Chau JY, van der Ploeg HP, Merom D, Chey T, Bauman AE. Cross-sectional associations between occupational and leisure-time sitting, physical activity and obesity in working adults. *Prev Med.* Mar-Apr 2012;54(3-4):195-200.

4. Du H, Bennett D, Li L, et al. Physical activity and sedentary leisure time and their associations with BMI, waist circumference, and percentage body fat in 0.5 million adults: the China Kadoorie Biobank study. *Am J Clin Nutr.* Mar 2013;97(3):487-496.

5. Dunton GF, Berrigan D, Ballard-Barbash R, Graubard B, Atienza AA. Joint associations of physical activity and sedentary behaviors with body mass index: results from a time use survey of US adults. *Int J Obes (Lond).* Dec 2009;33(12):1427-1436.

6. George ES, Rosenkranz RR, Kolt GS. Chronic disease and sitting time in middle-aged Australian males: findings from the 45 and Up Study. *Int J Behav Nutr Phys Act.* 2013;10:20.

7. Matthews CE, George SM, Moore SC, et al. Amount of time spent in sedentary behaviors and cause-specific mortality in US adults. *Am J Clin Nutr.* Feb 2012;95(2):437-445.

8. Craft LL, Zderic TW, Gapstur SM, et al. Evidence that women meeting physical activity guidelines do not sit less: an observational inclinometry study. *Int J Behav Nutr Phys Act.* 2012;9:122.

9. Dunstan DW, Howard B, Healy GN, Owen N. Too much sitting--a health hazard. *Diabetes Res Clin Pract.* Sep 2012;97(3):368-376.

10. Healy GN, Dunstan DW, Salmon J, et al. Breaks in sedentary time: beneficial associations with metabolic risk. *Diabetes Care.* Apr 2008;31(4):661-666.

11. Healy GN, Matthews CE, Dunstan DW, Winkler EA, Owen N. Sedentary time and cardio-metabolic biomarkers in US adults: NHANES 2003-06. *Eur Heart J.* Mar 2011;32(5):590-597.

12. Dunstan DW, Kingwell BA, Larsen R, et al. Breaking up prolonged sitting reduces postprandial glucose and insulin responses. *Diabetes Care.* May 2012;35(5):976-983.

13. Peddie MC, Bone JL, Rehrer NJ, Skeaff CM, Gray AR, Perry TL. Breaking prolonged sitting reduces postprandial glycemia in healthy, normal-weight adults: a randomized crossover trial. *Am J Clin Nutr.* Aug 2013;98(2):358-366.

14. Hamilton MT, Hamilton DG, Zderic TW. Role of low energy expenditure and sitting in obesity, metabolic syndrome, type 2 diabetes, and cardiovascular disease. *Diabetes.* Nov 2007;56(11):2655-2667.
15. Zderic TW, Hamilton MT. Identification of hemostatic genes expressed in human and rat leg muscles and a novel gene (LPP1/PAP2A) suppressed during prolonged physical inactivity (sitting). *Lipids Health Dis.* 2012;11:137.
16. Newgard CB. Interplay between lipids and branched-chain amino acids in development of insulin resistance. *Cell Metab.* May 2 2012;15(5):606-614.
17. Thalacker-Mercer AE, Ingram KH, Guo F, Ilkayeva O, Newgard CB, Garvey WT. BMI, RQ, Diabetes, and Sex Affect the Relationships Between Amino Acids and Clamp Measures of Insulin Action in Humans. *Diabetes.* Feb 2014;63(2):791-800.
18. Wang TJ, Larson MG, Vasan RS, et al. Metabolite profiles and the risk of developing diabetes. *Nat Med.* Apr 2011;17(4):448-453.
19. Wang TJ, Ngo D, Psychogios N, et al. 2-Aminoadipic acid is a biomarker for diabetes risk. *J Clin Invest.* Oct 1 2013;123(10):4309-4317.
20. Sears DD, Hsiao G, Hsiao A, et al. Mechanisms of human insulin resistance and thiazolidinedione-mediated insulin sensitization. *Proc Natl Acad Sci U S A.* Nov 3 2009;106(44):18745-18750.
21. Hsiao G, Chapman J, Ofrecio JM, et al. Multi-tissue, selective PPARgamma modulation of insulin sensitivity and metabolic pathways in obese rats. *Am J Physiol Endocrinol Metab.* Jan 2011;300(1):E164-174.
22. Dumas ME, Kinross J, Nicholson JK. Metabolic phenotyping and systems biology approaches to understanding metabolic syndrome and fatty liver disease. *Gastroenterology.* Jan 2014;146(1):46-62.
23. Newgard CB, An J, Bain JR, et al. A branched-chain amino acid-related metabolic signature that differentiates obese and lean humans and contributes to insulin resistance. *Cell Metab.* Apr 2009;9(4):311-326.
24. Thosar SS, Bielko SL, Mather KJ, Johnston JD, Wallace JP. Effect of Prolonged Sitting and Breaks in Sitting Time on Endothelial Function. Med Sci Sport Exerc. 2014:1. doi:10.1249/MSS.0000000000000479.
25. Restaino RM, Holwerda SW, Credeur DP, Fadel PJ, Padilla J. Impact of prolonged sitting on lower and upper limb micro- and macrovascular dilator function. Exp Physiol. 2015. doi:10.1113/EP085238.
26. Thosar SS, Johnson BD, Johnston JD, Wallace JP. Sitting and endothelial dysfunction: the role of shear stress. Med Sci Monit. 2012;18(12):RA173-RA180.
27. Thosar SS, Bielko SL, Wiggins CC, Wallace JP. Differences in brachial and femoral artery responses to prolonged sitting. 2014:1-7.
28. Saunders TJ, Larouche R, Colley RC, Tremblay MS. Acute sedentary behaviour and markers of cardiometabolic risk: A systematic review of intervention studies. J Nutr Metab. 2012;2012. doi:10.1155/2012/712435.
29. Larsen RN, Kingwell BA, Sethi P, Cerin E, Owen N, Dunstan DW. Breaking up prolonged sitting reduces resting blood pressure in overweight/obese adults. Nutr Metab Cardiovasc Dis. 2014;24(9):976-982.
30. Hamburg NM, McMackin CJ, Huang AL, et al. Physical inactivity rapidly induces insulin resistance and microvascular dysfunction in healthy volunteers. Arterioscler Thromb Vasc Biol. 2007;27(12):2650-2656.
31. Vernikos J, Schneider VS. Space, gravity and the physiology of aging: parallel or convergent disciplines? A mini-review. Gerontology. 2010;56(2):157-166.
32. Kerr, Jacqueline, Takemoto, Michelle, Bolling, Khalisa, Atkin, Andrew, Carlson, Jordan, Rosenberg, Dori, Crist, Katie, Godbole, Suneeta, Lewars, Brittany, Pena, Claudia, Merchant G.

Two-arm randomized pilot intervention to decrease sitting time and increase sit-to-stand transitions in working and non-working older adults. J Health Commun.
33. Bellettiere, J., Carlson, J.A., Rosenberg, D., Singhania, A., Natarajan, L., Berardi, V., LaCroix, A.Z., Sears, D.D., Moran, K., & Crist, K., Kerr, J. (2015). Gender and age differences in hourly and daily patterns of sedentary time in older adults living in retirement communities. PLoS ONE 10(8): e0136161. PMID: 26296095
34. American Diabetes Association. (6) Glycemic targets. *Diabetes Care*. Jan 2015;38 Suppl:S33-40.
35. American Diabetes Association. (10) Older adults. *Diabetes Care*. Jan 2015;38 Suppl:S67-69.

23. FUNDING SUPPORT FOR THIS STUDY

Funding for this pilot study comes from the Department of Family Medicine and Public Health Women's Health Center of Excellence. This study is intended to provide preliminary data for a Program Project proposal that we are submitting through the Department to the National Institute of Aging this year.

24. BIOLOGICAL MATERIALS TRANSFER AGREEMENT

N/A

25. INVESTIGATIONAL DRUG FACT SHEET AND IND/IDE HOLDER

N/A

26. IMPACT ON STAFF

CTRI CCR staff will obtain vital signs, instruct participants on the collection of urine, and perform phlebotomy, anthropomorphic measurements, FMD, and meal tolerance tests.

27. CONFLICT OF INTEREST

None.

28. SUPPLEMENTAL INSTRUCTIONS FOR CANCER-RELATED STUDIES

N/A

29. OTHER APPROVALS/REGULATED MATERIALS

N/A

30. PROCEDURES FOR SURROGATE CONSENT AND/OR DECISIONAL CAPACITY ASSESSMENT

N/A
